# Inhibition of lnc-COL6A1-6-Alleviated Osteogenic Differentiation of Valvular Interstitial Cells During Aortic Valve Calcification

**DOI:** 10.1155/cdr/2277191

**Published:** 2025-09-03

**Authors:** Ying Gu, Fan Yang, Quangong Zhao, Jingwen Zhou, Xiangyang Xu, Yang Yuan, Xian Guo, Zhigang Song, Zhiyun Xu, Guokun Wang

**Affiliations:** ^1^Department of Cardiology, Jinling Hospital, Nanjing University School of Medicine, Nanjing, Jiangsu, China; ^2^Department of Cardiovascular Surgery, Changhai Hospital, Naval Medical University, Shanghai, China; ^3^Department of Cardiothoracic Surgery, Nanjing Drum Tower Hospital, Affiliated Hospital of Medical School, Nanjing University, Nanjing, Jiangsu, China; ^4^Department of Respiratory and Critical Care Medicine, Tangdu Hospital, Air Force Medical University, Xi'an, Shaanxi, China

**Keywords:** autophagy, calcific aortic valve disease, long noncoding RNAs, osteogenic differentiation, valvular interstitial cells

## Abstract

**Background:** Calcific aortic valve disease (CAVD) is a prevalent valvular heart disease characterized by the fibrocalcific remodeling of the aortic valves, leading to significant health issues among the elderly population worldwide. The aberrant expression of long noncoding RNAs (lncRNAs) is closely associated with the pathogenesis of various diseases.

**Methods and Results:** A total of 241 differentially expressed lncRNAs were identified in calcified aortic valve tissues (fold change of ≥ 2 and *p* value < 0.05), including 65 upregulated and 176 downregulated lncRNAs. The expression of the Top 5 upregulated lncRNAs was monitored during the calcification of valvular interstitial cell (VIC). Notably, the expression of lnc-PRDM8-3 and lnc-COL6A1-6 in VICs increased significantly after calcification induction and was sustained at high levels. Inhibition of lnc-COL6A1-6, but not lnc-PRDM8-3, obviously alleviated the calcification of VICs, as evidenced by a marked reduction in calcium deposition, decreased alkaline phosphatase activity, and downregulated expression of Runx2 and OPN. Bioinformatic analysis predicted that lnc-COL6A1-6 might serve as a competing endogenous RNA for 11 miRNAs, potentially regulating the expression of 784 target genes. Among these, the Top 50 target genes were found to be significantly enriched in autophagy-related biological processes. Consistently, elevated levels of the autophagic markers Beclin 1 and LC3*β* were detected in calcified aortic valve tissues. Inhibition of lnc-COL6A1-6 significantly reduced autophagic flux in VICs under calcification-inducing conditions. Importantly, pharmacological inhibition of autophagy using chloroquine abolished the anticalcific effects of lnc-COL6A1-6 knockdown.

**Conclusions:** The present study identified a lnc-COL6A1-6-mediated miRNA–mRNA regulatory network in aortic valve calcification. Knockdown of lnc-COL6A1-6 could mitigate VIC calcification by attenuating autophagic activity, highlighting its potential as a therapeutic target for CAVD.

## 1. Introduction

Calcific aortic valve disease (CAVD) is the most common form of degenerative valvular disease, with an increasing prevalence observed in individuals over the age of 65 [1, 2]. The majority of CAVD patients initially present with aortic valve sclerosis and normal valve function, often being asymptomatic at the early stage. However, approximately 10% of these patients with sclerosis may progress to aortic valve stenosis within a 5-year period [3, 4]. Aortic valve calcification is a pathological process characterized by the deposition of calcium and phosphate in the aortic valve leaflets, which can lead from the initial aortic valve sclerosis to a more severe aortic valve stenosis [5]. Currently, surgical intervention and catheter-based therapies are the primary treatments aimed at alleviating symptoms and extending life expectancy [6]. Nonetheless, effective pharmacological therapies to control the pathogenesis and progression of CAVD have yet to be developed.

The pathogenesis of aortic valve calcification is a complex and dynamic process involving multiple regulated molecular mechanisms. These include the disruption of the endothelial layer, inflammation-induced calcification of valvular interstitial cells (VICs), oxidative stress, and dysregulation of lipid metabolism [7, 8]. VICs, as the primary cellular constituents of the aortic valve, play a crucial role in maintaining its structural integrity and functional efficiency, including the regulation of extracellular matrix composition and the response to mechanical stress [9, 10]. Increasing evidence suggests that the mineralization and transdifferentiation of VICs lead to extracellular matrix remodeling and calcium deposition, which ultimately contribute to the progression of CAVD [11, 12]. Therefore, elucidating the molecular mechanisms underlying the calcification of VICs could deepen our understanding of the development of CAVD and provide valuable insights for exploring potential therapeutic strategies.

Long noncoding RNAs (lncRNAs), which are sequences longer than 200 nucleotides and lack protein-coding capacity, can modulate a variety of molecular processes by influencing gene transcription and translation mechanisms [13]. Studies have revealed the close associations between aberrant lncRNA expression and the pathogenesis of human diseases [14, 15]. Our previous work also confirmed the abnormal expression patterns of lncRNAs in conditions such as cardiac hypertrophy, thoracic aortic aneurysm, and dissection [16–18]. More recently, a number of lncRNAs have been reported to play a role in the regulatory mechanisms associated with VIC calcification during the development of CAVD [19, 20]. The present study is aimed at identifying differentially expressed lncRNAs in calcified aortic valve tissues and exploring the role and potential mechanisms of lnc-COL6A1-6 in the calcification process of VICs.

## 2. Material and Methods

### 2.1. Clinical Samples

Calcified aortic valve leaflets were obtained from patients with CAVD who had undergone valve replacement at the Department of Cardiovascular Surgery. Noncalcified aortic valves were collected from the explanted hearts of patients undergoing heart transplant procedures. The exclusion criteria included bicuspid aortic valves, valves with moderate to severe aortic valve regurgitation, infective endocarditis, congenital valve disease, and rheumatic heart disease. The samples were either snap-frozen in liquid nitrogen for RNA analysis or fixed with 4% paraformaldehyde for histological analysis. The study conformed to the principles outlined in the Declaration of Helsinki. Written informed consent was obtained from all the participants before enrollment.

### 2.2. Histological Analysis

The collected aortic valve leaflets were embedded in paraffin and sectioned into 4–6 *μ*m slices. Following deparaffinization in xylene, the sections were rehydrated through a graded ethanol series. Subsequently, Alizarin Red S staining (Beyotime, Shanghai, China) and von Kossa staining (Solarbio, Beijing, China) were conducted for the evaluation of valve calcification, in accordance with the instruction manual. Immunohistochemistry assays were carried out for the detection of target proteins as described previously [9]. Details regarding the primary antibodies used are presented in Table S1.

### 2.3. Screening of Differentially Expressed lncRNAs

Differentially expressed lncRNAs between normal and calcified aortic valve tissues were identified based on the GSE233819 dataset in the Gene Expression Omnibus (GEO) database, with a cutoff for significance set at a fold change of ≥ 2 and a *p* value < 0.05. The LNCipedia database was subsequently applied to verify the information of differentially expressed lncRNAs [21].

### 2.4. Isolation of Primary Aortic VICs

Primary aortic VICs were isolated from normal aortic valve tissues as previously reported [10]. Briefly, aortic valve leaflets were enzymatically digested with Collagenase I (Sigma-Aldrich, St. Louis, MO, United States) for 30 min to facilitate the subsequent scraping removal of valvular endothelial cells. Following a 6-h redigestion with Collagenase I, the harvested cells were maintained in M199 medium (Thermo Fisher Scientific, Plainville, MA, United States) supplemented with 10% fetal bovine serum in a humidified atmosphere of 5% CO_2_ at 37°C. The purity of VICs, characterized by positivity for vimentin (Proteintech, Wuhan, China) and negativity for CD31 (Proteintech), was ascertained by immunofluorescence assay.

### 2.5. Calcification Induction of VICs

In vitro calcification of VICs was induced through a 6-day culture in an osteogenic medium, which consisted of M199 medium supplemented with 50 *μ*g/mL ascorbic acid, 2 mmol/L sodium dihydrogen phosphate dihydrate, and 0.1 *μ*mol/L insulin [22]. In a classical 14-day protocol, calcification was induced using M199 medium supplemented with 10 mmol/L *β*-glycerophosphate, 50 *μ*g/mL ascorbic acid, and 100 nmol/L dexamethasone [23]. The evaluation of calcification in VICs was conducted using Alizarin Red S staining to visualize mineralization, quantification of calcium content, and measurement of alkaline phosphatase (ALP) activity.

### 2.6. Small Interfering RNA (siRNA) Transfection

The siRNAs targeting lnc-PRDM8-3 (si-lnc-PRDM8-3: 5⁣′-GGAUUACAUCUCUGUGUUACA-3⁣′) and lnc-COL6A1-6 (si-lnc-COL6A1-6: 5⁣′-GGUAGAAACAGCACACGUAAG-3⁣′) were designed utilizing the DSIR algorithm [24]. These siRNAs and negative control (si-NC) were synthesized by Ribo Biotechnology Co. Ltd. Transfection of siRNAs was performed using the RiboFECT CP Transfection Kit (Ribo Biotechnology), following the manufacturer's protocol.

### 2.7. Quantitative Real-Time PCR (qRT-PCR)

Total RNA was extracted from aortic valve tissues or VICs using the MiniBEST Universal RNA Extraction Kit (Takara, Dalian, China), following the manufacturer's instructions. An equal amount (200 ng) of RNA was used as the template for reverse transcription to synthesize complementary DNA (cDNA) using the PrimeScript RT Reagent Kit (Takara). qRT-PCR was conducted in optical 96-well plates using TB Green qPCR Master Mix (Takara) and a Step One Real-Time PCR System (Roche, Basel, Switzerland). The relative expression of target lncRNAs or genes was calculated by using the 2^−*ΔΔ*Ct^ method, with GAPDH serving as the internal control. Primer sequences are listed in Table S2.

### 2.8. Western Blot

Total protein was extracted from VICs using RIPA lysis buffer plus cocktail (Thermo Fisher Scientific), and protein concentration was quantified by the bicinchoninic acid assay (Beyotime). Equal quantities of proteins were separated by 10% sodium dodecyl sulfate polyacrylamide gel electrophoresis (SDS-PAGE). Subsequently, the proteins were transferred onto a polyvinylidene fluoride (PVDF) membrane (Millipore, Billerica, MA, United States) and blocked with 5% nonfat milk in Tris-buffered saline with 0.5% Tween 20. The membranes were then probed with the indicated primary antibodies overnight at 4°C, followed by incubation with the corresponding horseradish peroxidase–conjugated secondary antibodies for 1 h at room temperature. The immunoblots were finally visualized using ECL Plus Western Blotting Substrate (Thermo Fisher Scientific) on a ChemiDoc MP system (Bio-Rad, Hercules, CA, United States). The density of immunoblots was quantified by ImageJ software (National Institutes of Health, Bethesda, MD, United States). GAPDH was used as a loading control.

### 2.9. Bioinformatic Analysis

The potential interactions between lncRNAs and miRNAs, as well as the prediction of miRNA target genes, were analyzed using the miRDB database [25]. Enrichment analyses of the identified genes, encompassing Gene Ontology (GO), Kyoto Encyclopedia of Genes and Genomes (KEGG) pathways, and Reactome pathways, were performed using the OECloud tools available at https://cloud.oebiotech.com.

### 2.10. Luciferase Reporter Assay

The 3× repeating sequences of lnc-COL6A1-6 were synthesized and subcloned into a luciferase reporter plasmid as the 3⁣′-untranslated regions of the luciferase gene. The recombinant reporter plasmid (pGL3-lnc-COL6A1-6), a Renilla reference plasmid, and miRNA mimic were cotransfected into HEK293T cells using Lipofectamine 3000 (Thermo Fisher Scientific). Luciferase activities were measured 48 h posttransfection using a luciferase reporter assay kit (Beyotime) according to the manufacturer's instructions.

### 2.11. Autophagic Flux Monitoring

Autophagic flux in VICs was assessed using the mRFP-GFP-LC3B reporter system. Briefly, VICs were transfected with adenovirus expressing mRFP-GFP-LC3B (Hanbio Biotechnology, Shanghai, China) and then subjected to the indicated treatments. Following fixation with 4% paraformaldehyde, autophagic flux was evaluated by monitoring the differential stability of GFP and mRFP fluorescence signals.

### 2.12. Statistical Analysis

Data were analyzed using SPSS 21.0 software. Continuous data are presented as mean ± standard deviation (SD). For continuous, normally distributed data with equal variances, comparisons were made using Student's *t*-test or one-way ANOVA followed by Bonferroni post hoc tests when necessary. Nonnormally distributed variables were analyzed using the Mann–Whitney *U* test. A *p* value of < 0.05 was considered statistically significant.

## 3. Results

### 3.1. Differentially Expressed lncRNAs in Calcified Aortic Valve Tissues

**Figure 1:**
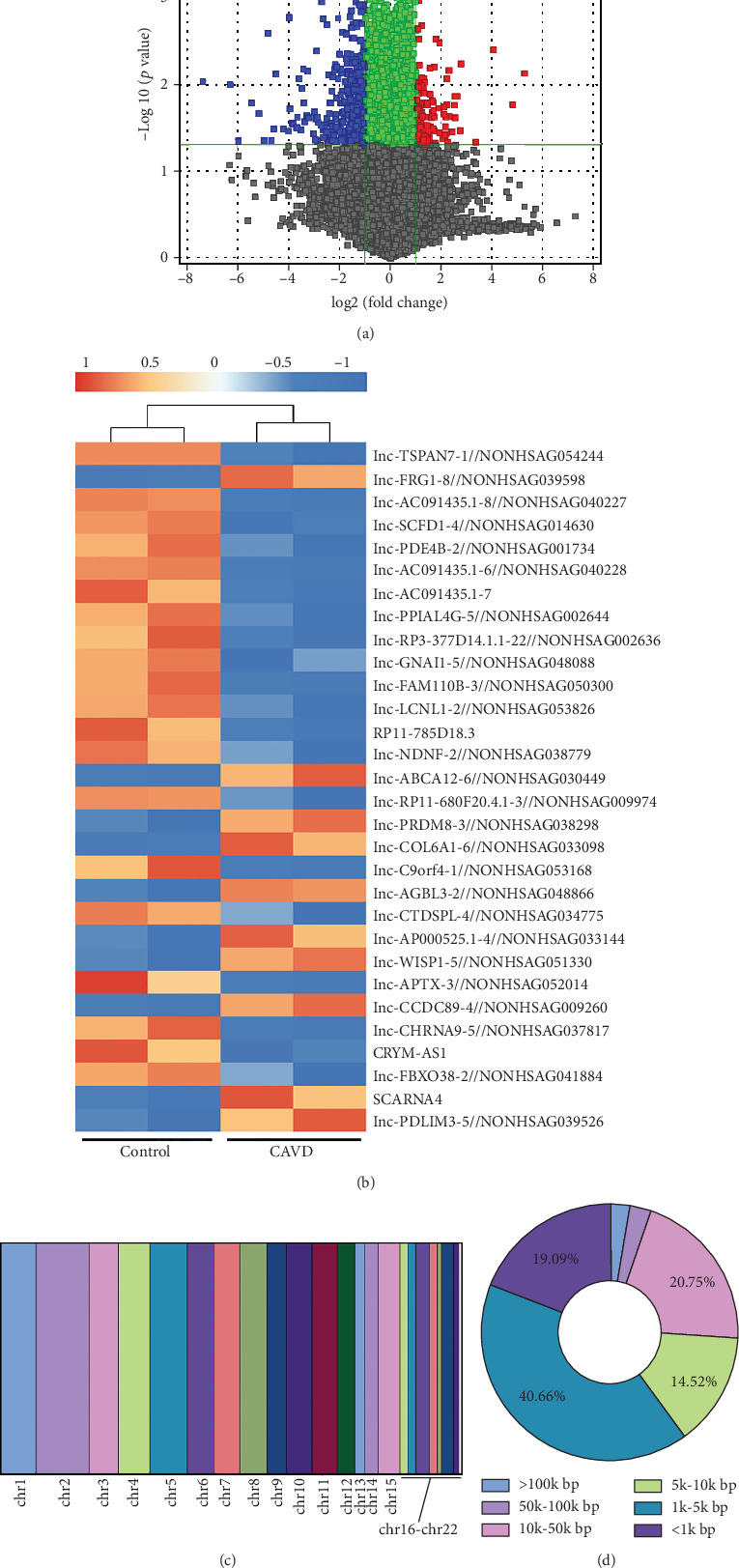
Differentially expressed lncRNAs in calcified aortic valve tissues. (a) Volcano plots depicted the differentially expressed lncRNAs with significant differential expression. Red and blue points indicated lncRNAs with a fold change ≥ 2 and a *p* value < 0.05, respectively. Green points represented lncRNAs with a fold change < 2 and a *p* < 0.05, and gray points represented lncRNAs significant at the *p* value < 0.05 level. (b) Hierarchical clustering displayed the Top 30 differentially expressed lncRNAs. Red indicated relatively high expression, while green indicated relatively low expression. (c) Chromosome locations of the differentially expressed lncRNAs. (d) Distribution of nucleotide length for the differentially expressed lncRNAs.

Our previous study conducted a comparative analysis of the mRNA expression profile between normal and calcified aortic valve tissues, identifying a set of differentially expressed transcripts that may contribute to the pathogenesis of aortic valve calcification [9]. Utilizing data from the GSE233819 dataset and the LNCipedia database, a total of 241 lncRNAs were detected as differentially expressed in the CAVD group compared to the control group (fold change of ≥ 2 and *p* value < 0.05), including 65 upregulated and 176 downregulated lncRNAs (Figure 1a). The Top 30 differentially expressed lncRNAs were visualized using heat maps, as shown in Figure 1b. Subsequently, we analyzed the genomic locations and transcript lengths of these lncRNAs. The majority of the dysregulated lncRNAs were found on autosomes, with Chromosome 2 being the most represented (Figure 1c). In terms of length, these lncRNAs were predominantly shorter than 10 kilobases (kb), with approximately 50% ranging from 1 to 5 kb (Figure 1d).

### 3.2. Multisample Validation of lncRNA Expression Profiles by qRT-PCR

**Figure 2:**
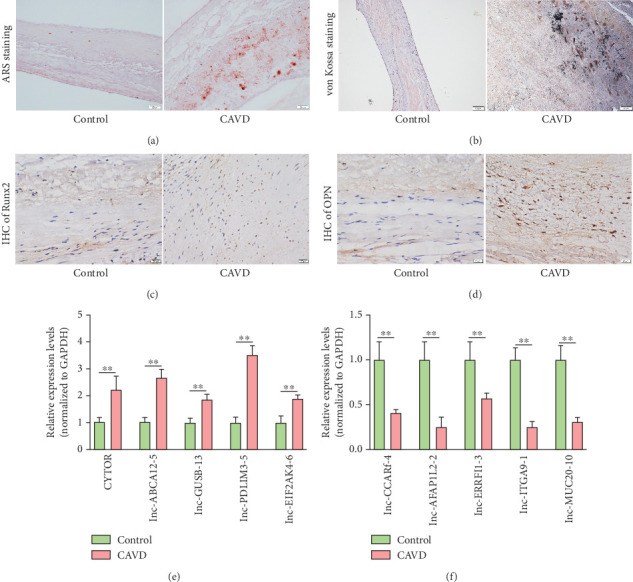
Multisample validation of lncRNA expression profiles by qRT-PCR. (a, b) Representative images of Alizarin Red S (ARS) staining and von Kossa staining in paraffin sections of aortic valve tissues. (a) Red and (b) black signals indicated the mineral deposition. Scale bar = 50 *μ*m. (c, d) Representative images of immunohistochemistry (IHC) detection for Runx2 and OPN in paraffin sections of aortic valve tissues. Scale bar = 20 *μ*m. (e, f) qRT-PCR validation of 10 randomly selected lncRNAs in aortic valve tissues from the control (*n* = 15) and CAVD (*n* = 20) groups. ⁣^∗∗^*p* < 0.01.

To validate the microarray data, a total of 35 aortic valve tissues, including 15 normal and 20 mild or moderate calcified aortic valve samples, were collected in this study. No significant difference in baseline characteristics were found between control and CAVD groups (Table 1). Alizarin Red S staining and von Kossa staining revealed few calcified nodules or mineral deposition in aortic valve tissues from the control group. In contrast, significantly increased calcified nodules were detected in the mild or moderate calcified aortic valves from CAVD patients compared to normal subjects (Figure 2a,b). Additionally, the expression of osteogenic markers OPN and Runx2 was markedly elevated in calcified aortic valve tissues, as confirmed by immunohistochemistry assays (Figure 2c,d). Ten lncRNAs (five upregulated and five downregulated) were randomly selected and analyzed in normal and calcified aortic valve tissues using qRT-PCR. The expression levels of CYTOR, lnc-ABCA12-5, lnc-GUSB-13, lnc-PDLIM3-5, and lnc-EIF2AK4-6 were significantly increased in calcified aortic valves, while the expression of lnc-CCAR1-4, lnc-AFAP1L2-2, lnc-ERRFI1-3, lnc-ITGA9-1, and lnc-MUC20-10 was decreased in the CAVD group (Figure 2e,f).

### 3.3. Detection of the Top 5 Upregulated lncRNAs During VIC Calcification

**Figure 3:**
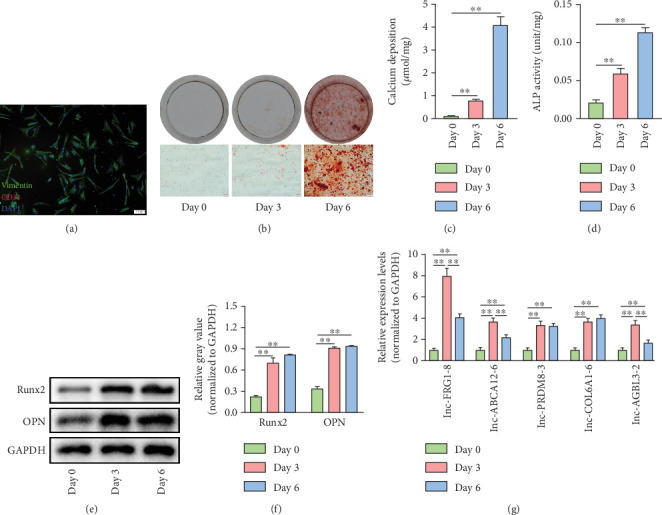
Detection of the Top 5 upregulated lncRNAs during VIC calcification. (a) Representative images of immunofluorescence for vimentin (green) and CD31 (red) in primary isolated VICs. Scale bar = 100 *μ*m. (b) Representative images of Alizarin Red S (ARS) staining for mineral deposition in VICs after calcification induction. The lower panel shows the optical microscope images with magnification. Scale bar = 100 *μ*m. (c) Quantification of the calcium concentration in VICs after calcification induction. ⁣^∗∗^*p* < 0.01. (d) Determination of ALP activity in VICs after calcification induction. ⁣^∗∗^*p* < 0.01. (e, f) Representative images of western blot assay for Runx2 and OPN protein expression during VIC calcification. Relative gray analysis of immune bands was quantified using ImageJ software. *n* = 4 in each group. ⁣^∗∗^*p* < 0.01. (g) qRT-PCR detection of the Top 5 upregulated lncRNAs during VIC calcification. *n* = 4 in each group. ⁣^∗∗^*p* < 0.01.

In the ongoing quest to unravel the molecular underpinnings of valvular calcification, we focused on the Top 5 upregulated lncRNAs (lnc-FRG1-8, lnc-ABCA12-6, lnc-PRDM8-3, lnc-COL6A1-6, and lnc-AGBL3-3) and detected their expression profiles during the osteogenic differentiation of VICs. Primary VICs were isolated from noncalcified aortic valve tissues and identified using immunofluorescence for vimentin and CD31. The purity of the isolated cell population was high, with more than 95% of the cells expressing vimentin and lacking CD31 (Figure 3a). Subsequently, VICs were cultured in an osteogenic medium for a designated period to induce calcification. Continuous exposure to this medium resulted in a significant increase in the formation of mineral nodules and calcium deposition in VICs, as evidenced by Alizarin Red S staining assay (Figure 3b,c), and was accompanied by a significant increase in ALP activity (Figure 3d). Furthermore, western blot assay confirmed that the expression of Runx2 and OPN was significantly increased in VICs after 3- or 6-day osteogenic induction, respectively, indicating the activation of osteogenic signaling (Figure 3e,f). Additionally, qRT-PCR assay showed that the expression of the five lncRNAs was significantly increased in VICs exposed to the osteogenic medium (Figure 3f). Notably, the expression of lnc-FRG1-8, lnc-ABCA12-6, and lnc-AGBL3-2 was reduced on Day 6 compared to Day 3, while the expression of lnc-PRDM8-3 and lnc-COL6A1-6 remained at a high level (Figure 3f). Elevated expression of the five lncRNAs was also observed under an alternative calcification induction protocol (Figure S1).

### 3.4. Inhibition of lnc-COL6A1-6 Alleviated the Calcification of VICs

**Figure 4:**
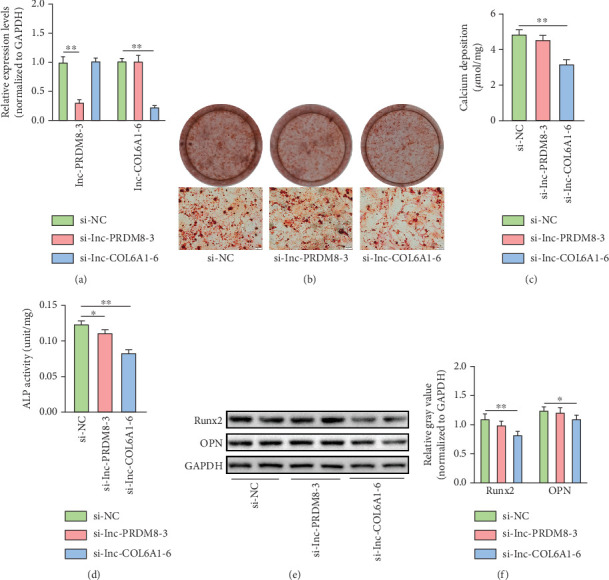
Inhibition of lnc-COL6A1-6 alleviated the calcification of VICs. (a) qRT-PCR detection of lnc-PRDM8-3 and lnc-COL6A1-6 expression in VICs after siRNA transfection. *n* = 4 in each group. ⁣^∗∗^*p* < 0.01. (b) Representative images of Alizarin Red S staining for mineral deposition in VICs after siRNA transfection under conditions of calcification induction. The lower panel shows the optical microscope images with magnification. Scale bar = 100 *μ*m. (c) Quantification of the calcium concentration in VICs after siRNA transfection. ⁣^∗∗^*p* < 0.01. (d) Determination of ALP activity in VICs after calcification induction. ⁣^∗^*p* < 0.05 and ⁣^∗∗^*p* < 0.01. (e, f) Representative images of western blot assay for Runx2 and OPN protein expression in VIC with siRNA transfection. Relative gray analysis of immune bands was quantified using ImageJ software. *n* = 4 in each group. ⁣^∗^*p* < 0.05 and ⁣^∗∗^*p* < 0.01.

Analyzing the expression pattern of these lncRNAs in the calcification process of VICs, we conducted loss-of-function experiments utilizing siRNA to target lnc-PRDM8-3 or lnc-COL6A1-6. qRT-PCR assays confirmed that transfection of siRNA significantly decreased the expression levels of the targeted lncRNA (Figure 4a). After a 6-day osteogenic induction, VICs transfected with siRNA specifically targeting lnc-COL6A1-6 (si-lnc-COL6A1-6) exhibited a significant reduction in mineral nodule count (Figure 4b), decreased calcium deposition (Figure 4c), lowered ALP activity (Figure 4d), and diminished expression of Runx2 and OPN (Figure 4e,f), as compared to those transfected with nontargeting siRNA (si-NC). However, inhibition of lnc-PRDM8-3 did not significantly affect these markers of VIC calcification, except for a reduction in ALP activity (Figures 4b, 4c, 4d, 4e, and 4f).

### 3.5. Regulation of lnc-COL6A1-6-miRNA–mRNA Network on VIC Autophagy

**Figure 5:**
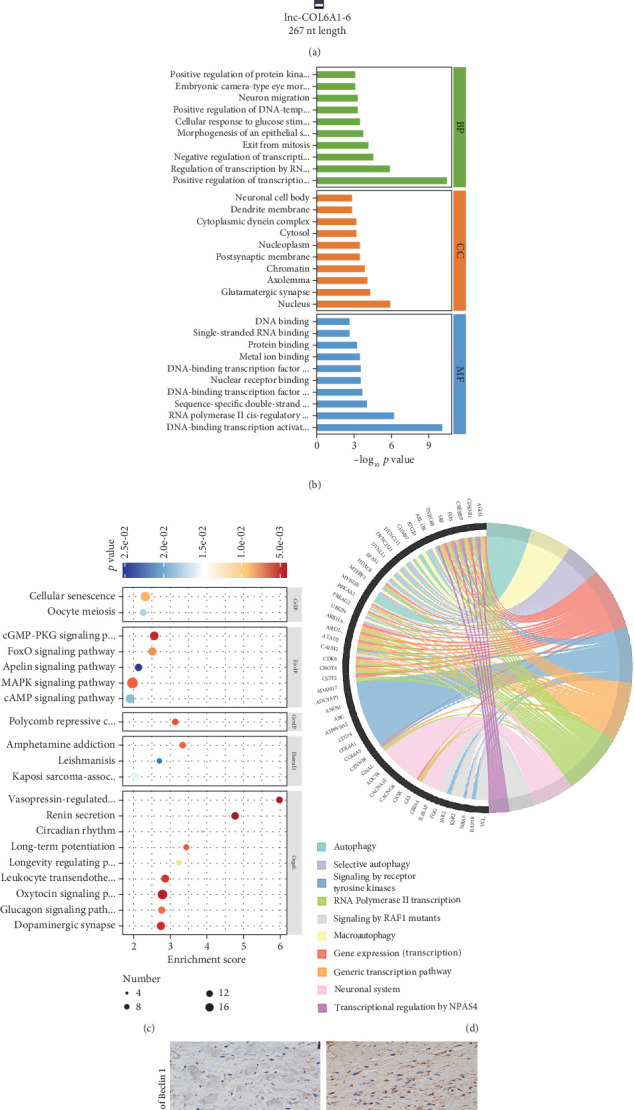
Regulation of lnc-COL6A1-6-miRNA–mRNA network on VIC autophagy. (a) The schematic diagram illustrated the potential interactions between lnc-COL6A1-6 and miRNAs. (b) GO enrichment analysis of the potential target genes of miRNAs interacting with lnc-COL6A1-6. Top 10 terms of enrichment in each ontology were listed. (c) KEGG enrichment analysis of these potential target genes. Top 20 terms of enrichment were listed. (d) Reactome enrichment analysis of the Top 50 potential target genes. (e, f) Representative images of immunohistochemistry (IHC) detection for Beclin 1 and LC3*β* in paraffin sections of aortic valve tissues. Scale bar = 20 *μ*m.

It is well-established that lncRNAs perform multiple biological functions by acting as competing endogenous RNAs (ceRNAs), thereby reducing the inhibitory effects of miRNAs on protein-coding target genes. Consequently, several bioinformatic algorithms were utilized to construct the lnc-COL6A1-6-mediated miRNA–mRNA regulatory networks. Bioinformatic analysis utilizing the miRDB database identified 11 miRNAs with putative binding sites to lnc-COL6A1-6, including miR-200a/b and miR-517a/b/c (Figure 5a). Subsequently, a total of 619 target genes and 784 miRNA–mRNA pairs were retrieved from the miRDB database (Table S3). GO functional analysis revealed that the majority of target genes were enriched in the regulation of transcription by RNA Polymerase II (positive or negative) in the biological process analysis. These target genes were related to nuclear localization in the cellular component analysis and correlated with protein binding and metal ion binding in the molecular function analysis (Figure 5b). KEGG enrichment analysis confirmed that these genes were significantly enriched in the MAPK signaling pathway and the cGMP-PKG signaling pathway (Figure 5c). Additionally, Reactome enrichment analysis of the Top 50 genes indicated that autophagy, macroautophagy, and selective autophagy emerged as the highly enriched pathways (Figure 5d). Immunohistochemistry assay also confirmed that the expression of the autophagy markers (Beclin 1 and LC3*β*) in aortic valve tissues from the CAVD group was significantly higher than that observed in the control group (Figure 5e,f). These results suggested the potential role of the lnc-COL6A1-6-miRNA–mRNA regulatory network on autophagy in the process of aortic valve calcification.

### 3.6. Silencing lnc-COL6A1-6-Mitigated VIC Calcification Through Attenuating Autophagy

**Figure 6:**
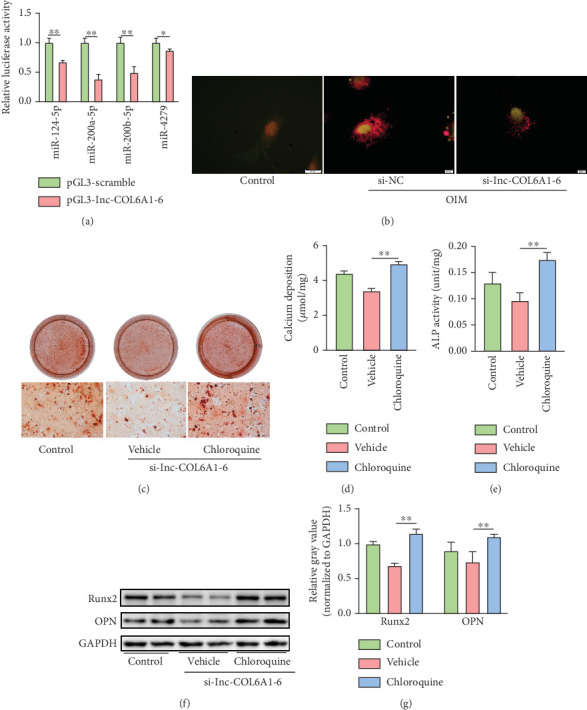
Autophagy mediated the anticalcific effects of lnc-COL6A1-6 knockdown. (a) A luciferase reporter assay confirmed the direct binding between miRNAs and lnc-COL6A1-6. ⁣^∗^*p* < 0.05 and ⁣^∗∗^*p* < 0.01. (b) Representative images of fluorescent-tagged autophagic flux in VICs following siRNA transfection under calcification conditions. (c) Representative images of Alizarin Red S staining for mineral deposition in chloroquine-pretreated VICs transfected with si-lnc-COL6A1-6. The lower panel shows the optical microscope images with magnification. Scale bar = 100 *μ*m. (d) Quantification of the calcium concentration in chloroquine-pretreated VICs transfected with si-lnc-COL6A1-6. ⁣^∗∗^*p* < 0.01. (e) Assessment of ALP activity in chloroquine-pretreated VICs transfected with si-lnc-COL6A1-6. ⁣^∗∗^*p* < 0.01. (f, g) Representative images of western blot assay for Runx2 and OPN protein expression in chloroquine-pretreated VICs transfected with si-lnc-COL6A1-6. Relative gray analysis of immune bands was quantified using ImageJ software. *n* = 4 in each group. ⁣^∗∗^*p* < 0.01.

Based on the enrichment analyses of the predicted miRNA targets, autophagy-related genes were predominantly enriched among the potential targets of miR-124-5p, miR-200a-5p, miR-200b-5p, and miR-4279 (Table S3). Luciferase reporter assays subsequently revealed that transfection with mimics of miR-124-5p, miR-200a-5p, miR-200b-5p, or miR-4279 significantly suppressed luciferase activity in constructs containing lnc-COL6A1-6 sequences (Figure 6a). To further investigate the role of lnc-COL6A1-6 in autophagy, the mRFP-GFP-LC3B reporter system was employed to monitor autophagic flux in VICs. Under calcification-inducing conditions, knockdown of lnc-COL6A1-6 resulted in a marked reduction in autophagic flux compared to the si-NC group (Figure 6b). To determine whether the anticalcific effects of lnc-COL6A1-6 knockdown were dependent on autophagy, VICs were pretreated with chloroquine (10 *μ*mol/L), a well-established inhibitor of autophagic flux, followed by si-lnc-COL6A1-6 transfection. The results showed that knockdown of lnc-COL6A1-6 had no significant anticalcific effect in VICs with impaired autophagy, as evidenced by calcium deposition levels (Figure 6c,d), ALP activity (Figure 6e), and expression of Runx2 and OPN (Figure 6f,g). These results suggested that knockdown of lnc-COL6A1-6 might mitigate VIC calcification through attenuating autophagic activity.

## 4. Discussion

In the present study, we identified differential expression profiles of lncRNAs in calcified aortic valve tissues and confirmed the elevated expression of lnc-COL6A1-6 in the calcification process of VICs and aortic valves. Inhibition of lnc-COL6A1-6 was found to alleviate the calcification of VICs, potentially through its regulation of autophagy via a miRNA–mRNA network. Although the specific components of this network require further investigation, our findings reveal a novel potential mechanism involving lncRNA-miRNA–mRNA interactions in aortic valve calcification.

The transition of VICs into osteoblast-like cells is recognized as a critical process in the development of CAVD. Although the precise mechanisms underlying this phenotypic transition remain elusive, transcriptomic analyses of both mRNA and noncoding RNA expression profiles have been explored in the context of VIC osteogenic differentiation or aortic valve calcification [26, 27]. Integrative expression profile analysis could potentially identify candidate causal target genes or biological processes involved in the pathogenesis of CAVD. Based on the differential gene expression, our previous study revealed that disruption of ribosomal homeostasis, caused by decreased expression of ribosomal proteins, might present a novel mechanism contributing to aortic valve calcification [9]. In the present study, it was found that lnc-COL6A1-6-mediated miRNA–mRNA network might be implicated in aortic valve calcification through the regulation of autophagic activity, offering a potential new avenue for understanding the pathogenesis of CAVD.

lnc-COL6A1-6, designated as NONHSAT083000.2 in the NONCODE database [28], is situated on the reverse strand of a region within human Chromosome 21, with positions spanning from 45,923,213 to 45,925,517. To date, no correlation between the expression of lnc-COL6A1-6 and any specific disease has been reported. In the present study, we observed the elevated expression of lnc-COL6A1-6 in calcified aortic valve tissues and confirmed that its inhibition could alleviate the calcification of VICs. Recently, the role of specific lncRNAs, including SNHG3 [19], LINC01013 [20], and lncTSI [29], in aortic valve calcification has been reported. These findings suggest the important regulatory roles of lncRNAs in the pathogenesis of CAVD.

Studies have indicated that lncRNAs participate in various physiological and pathological processes, including epigenetic modifications, transcriptional regulation, posttranscriptional events, translational control, and posttranslational modifications [30]. The regulatory mechanism, whereby lncRNAs act as miRNA sponges, sequestering miRNAs to modulate their activity, is a crucial component of the current understanding of lncRNA-mediated posttranscriptional regulation. Multiple lncRNA-mediated miRNA–mRNA networks, such as the TUG-miR-204-5p-Runx2 and MALAT1-miR-191-3p-HuR networks [31, 32], have been reported to be implicated in aortic valve calcification. In this study, a total of 11 miRNAs with putative binding sites to lnc-COL6A1-6 were identified by bioinformatic prediction. Although the target genes of these miRNAs remain to be confirmed, GO and KEGG pathway analyses suggested that lnc-COL6A1-6 might regulate autophagic activity in VICs through the intricate ceRNA crosstalk and competition mechanisms, potentially playing a role in the pathogenesis of CAVD.

Autophagy is a complex intracellular process that degrades dysfunctional cellular components, thereby maintaining cellular homeostasis [33, 34]. Alterations in autophagic activity have been closely associated with a variety of pathological processes, such as neurodegenerative diseases, cancer, and cardiovascular disorders [35, 36]. The present study confirmed increased autophagic activity in calcified aortic valve tissues, findings consistent with recent reports and suggesting a potential role of autophagy in the pathogenesis of CAVD. However, the precise role of autophagy in CAVD remains unclear, potentially due to its complex, double-edged sword effect. Several studies have found that activated autophagy in calcific aortic valves was insufficient to counteract cell death and sustain cell functions, suggesting that boosting autophagy might improve the pathological phenotypes in CAVD [37, 38]. In contrast, Fang et al. reported that silencing of human antigen R (HuR) ameliorated aortic valve calcification by suppressing autophagy, highlighting the potential of targeting HuR to inhibit autophagy as an innovative therapeutic strategy for CAVD [39]. Therefore, while our results indicated a potential regulatory role of lnc-COL6A1-6 in VIC autophagy, the underlying mechanisms and its role in the pathogenesis of CAVD require further investigation.

While this study provides valuable insights into the role of lnc-COL6A1-6 in CAVD, several limitations should be acknowledged. First, the findings rely primarily on an unconventional osteogenic differentiation model and therefore need to be validated using a classical differentiation protocol. Second, although siRNA-mediated knockdown effectively reduced lnc-COL6A1-6 expression, potential off-target effects cannot be ruled out and should be carefully evaluated. Third, although autophagy is implicated as a key underlying mechanism, the precise regulatory role of lnc-COL6A1-6 remains to be elucidated. Furthermore, the study lacks in vivo validation, which is essential to determine the translational potential of targeting lnc-COL6A1-6. Addressing these limitations through expanded experimental models, detailed mechanistic studies, and preclinical investigations will enhance the clinical relevance of these findings.

In conclusion, the current study has identified a lnc-COL6A1-6-mediated miRNA–mRNA regulatory network in the calcification process of VICs and aortic valves. Knockdown of lnc-COL6A1-6 mitigated VIC calcification by attenuating autophagic activity, positioning it as a promising therapeutic target for CAVD. While the specific molecular interactions within this network remain to be fully elucidated, ongoing research is actively investigating these mechanisms. Understanding these interactions could yield significant insights into the regulatory functions of lnc-COL6A1-6 in the context of CAVD.

## Figures and Tables

**Table 1:** General information and clinical characteristics of the enrolled subjects.

**Parameters**	**Control group (** **n** = 15**)**	**CAVD group (** **n** = 20**)**	**p** ** value**
Age (years)	67.0 ± 7.6	63.8 ± 9.4	0.289
Male, *n* (%)	7 (46.7)	11 (61.1)	0.407
BMI (kg/m^2^)	24.2 ± 2.5	23.4 ± 4.2	0.527
Smoking, *n* (%)	5 (33.3)	6 (30.0)	0.833
Hypertension, *n* (%)	5 (38.5)	11 (55.0)	0.353
DM, *n* (%)	4 (26.7)	5 (25.0)	0.911
Hyperlipidemia, *n* (%)	3 (20.0)	7 (35.0)	0.331
HDL-C (mmol/L)	1.25 ± 0.38	1.23 ± 0.27	0.854
LDL-C (mmol/L)	2.85 ± 0.97	2.75 ± 0.93	0.752
Triacylglycerols (mmol/L)	1.21 ± 0.55	1.43 ± 0.68	0.303
Creatine (*μ*mol/L)	78.67 ± 15.05	74.15 ± 20.86	0.483
LVEF (%)	60.47 ± 5.32	58.70 ± 7.27	0.433

Abbreviations: BMI, body mass index; DM, diabetes mellitus; HDL-C, high-density lipoprotein cholesterol; LDL-C, low-density lipoprotein cholesterol; LVEF, left ventricular ejection fraction.

## Data Availability

The data that support the findings of this study are openly available in Gene Expression Omnibus at https://www.ncbi.nlm.nih.gov/geo/, Reference Number GSE233819.

## Nomenclature

CAVD: calcific aortic valve disease
lncRNAs: long noncoding RNAs
VIC: valvular interstitial cell
GEO: Gene Expression Omnibus
ALP: alkaline phosphatase
siRNA: small interfering RNA
qRT-PCR: quantitative real-time PCR
cDNA: complementary DNA
SDS-PAGE: sodium dodecyl sulfate polyacrylamide gel electrophoresis
PVDF: polyvinylidene fluoride
GO: Gene Ontology
KEGG: Kyoto Encyclopedia of Genes and Genomes
SD: standard deviation
kb: kilobases
ceRNA: competing endogenous RNA

## Supporting Information

Supporting Information Additional supporting information can be found online in the Supporting Information section. Figure S1: Expression of the Top 5 upregulated lncRNAs in VICs following a classic 14-day calcification induction protocol. Table S1: The detailed application information of antibodies. Table S2: The sequences of primers used in the qRT-PCR assay. Table S3: The potential target genes of miRNAs predicted by the miRDB database.
