# Supplementary material for: Inhibition of lnc-COL6A1-6-Alleviated Osteogenic Differentiation of Valvular Interstitial Cells During Aortic Valve Calcification
Source: Cardiovasc Ther. 2025 Sep 3;2025:2277191. doi: 10.1155/cdr/2277191 (PMC12422864; doi:10.1155/cdr/2277191)
Supplement: Supporting Information — Additional supporting information can be found online in the Supporting Information section. Figure S1: Expression of the Top 5 upregulated lncRNAs in VICs following a classic 14-day calcification induction protocol. Table S1: The detailed application information of antibodies. Table S2: The sequences of primers used in the qRT-PCR assay. Table S3: The potential target genes of miRNAs predicted by the miRDB database. [file 2277191.f1.docx]

Supplemental Data


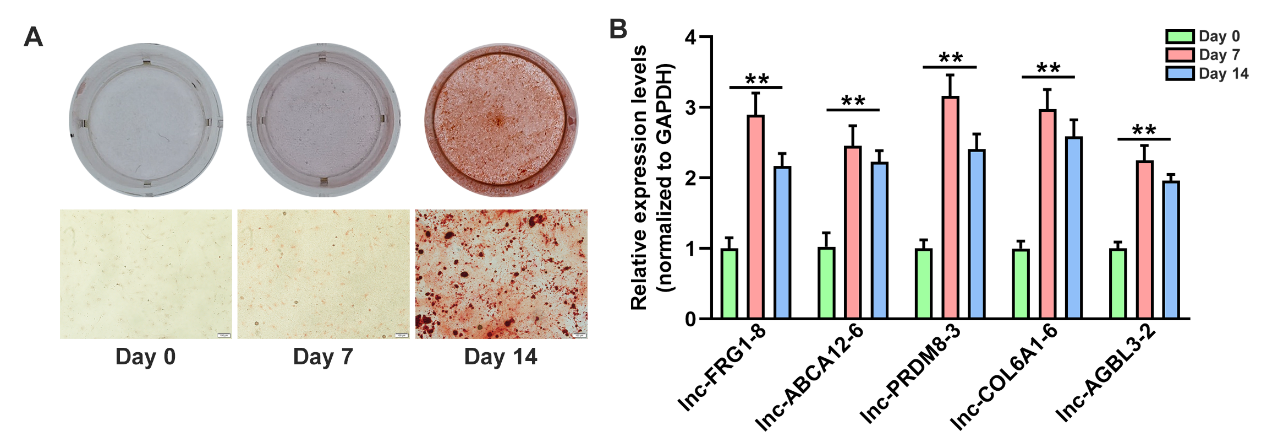


Figure S1 Expression of the top 5 up-regulated lncRNAs in VICs following 7- or 14-day calcification induction. A, Representative Alizarin Red S (ARS) staining images for mineral deposition in VICs after calcification induction. The lower panel displays the optical microscope images with magnification. Scale bar = 100 μm. B, qRT‐PCR analysis of the top 5 upregulated lncRNAs during calcification of VIC. n = 4 in each group. **P < 0.01.

Table S1 The detailed application information of antibodies

| Name | Description | Company | Application information |
| --- | --- | --- | --- |
| anti-OPN | Rabbit monoclonal | Abcam, Cambridge, UK | 1:1000 dilution in WB; 1:2000 dilution for IHC |
| anti-Runx2 | Rabbit monoclonal | Abcam, Cambridge, UK | 1:1000 dilution in WB;  1:1000 dilution for IHC |
| anti-vimentin | Rabbit monoclonal | Proteintech, Wuhan, China | 1:400 dilution for IF |
| anti-CD31 | Rabbit monoclonal | Proteintech, Wuhan, China | 1:200 dilution for IF |
| anti-GAPDH | Mouse monoclonal | Proteintech, Wuhan, China | 1:5000 dilution for WB |

Table S2 The sequences of primers used in the qRT-PCR assay

| **Name** | **Sequences (5’-3’)** |
| --- | --- |
| CYTOR | forward: CGTTCAGTTGGCAAGGTCAA; reverse: ACAGATTCCAGCTCAGACGT |
| lnc-ABCA12-5 | forward: ATAGGCCAAGACCATACCCG; reverse: AGAAGTGTCCTGGAATGGGG |
| lnc-GUSB-13 | forward: TGTTAGCTGAGATTGTGCCAC; reverse: TCCATTCCACTAGACTCCCC |
| lnc-PDLIM3-5 | forward: CAAGCCGGATGATGACACAG; reverse: TGTTGTCGGAGTGGCTTTTG |
| lnc-EIF2AK4-6 | forward: ACGGAAAGTGTTTGAGAGCAA; reverse: ATCCACTCTGAGCACAAGGG |
| lnc-CCAR1-4 | forward: ATCTGTTGTGGCTGGCTTTG; reverse: TCCTGGATTCTTTGCCTTGC |
| lnc-AFAP1L2-2 | forward: GGTACCAGGATGTTCGGGAT; reverse: CAGGTCTGTGGAAATGCTGG |
| lnc-ERRFI1-3 | forward: TTAACGCTGGCTGTGGAAAC; reverse: CATGTCAGCTTTGCCTCCAT |
| lnc-ITGA9-1 | forward: AGGATTGTCACTTGGAGCCT; reverse: TCCCTTCAGCCACTCTTTGA |
| lnc-MUC20-10 | forward: GAACGATCACTTGAGCCCAG; reverse: ATCTTCCCGACTCAGCCTTC |
| lnc-FRG1-8 | forward: CACCACCATCACCACATCACCAC; reverse: GGTGTGTGTGCTAGTGCAGTGG |
| lnc-ABCA12-6 | forward: ACCATCCCAGATGCAAGTGA; reverse: AACTTGAAGGCAGCCACTTG |
| lnc-PRDM8-3 | forward: GAACAGGAGGCTGACAGGAT; reverse: GTTAAACAAAGCCAGCCCTCT |
| lnc-COL6A1-6 | forward: TGCACGAGGAGATGCGAACAC; reverse: TGCTGTTTCTACCCACTCGACTG |
| lnc-AGBL3-2 | forward: ACCCTCATGACAAAGTAGGCA; reverse: GGAGTCAATGCTACCTGGGA |
| GAPDH | forward: ACCACCATGGAGAAGGCTG; reverse: GGTCATGAGTCCTTCCACGA |

Table S3 The potential target genes of miRNAs predicted by miRDB database

| miRNA_v22 | Gene Symbol | miRDB_mRNA | Score | miRNA_v22 | Gene Symbol | miRDB_mRNA | Score |
| --- | --- | --- | --- | --- | --- | --- | --- |
| hsa-miR-200a-5p | BTBD1 | NM_025238 | 99.76 | hsa-miR-200a-5p | ZNF99 | NM_001080409 | 97.11 |
| hsa-miR-200b-5p | BTBD1 | NM_025238 | 99.76 | hsa-miR-200b-5p | ZNF99 | NM_001080409 | 97.11 |
| hsa-miR-8064 | WDFY3 | NM_014991 | 99.45 | hsa-miR-4279 | PGPEP1 | NM_001308366 | 96.95 |
| hsa-miR-4279 | NFASC | NM_001160331 | 99.19 | hsa-miR-4279 | SRF | NM_001292001 | 96.92 |
| hsa-miR-4279 | RIMS3 | NM_014747 | 98.96 | hsa-miR-8064 | PGGT1B | NM_005023 | 96.85 |
| hsa-miR-200a-5p | SP3 | NM_001172712 | 98.53 | hsa-miR-4279 | GLRA4 | NM_001172285 | 96.84 |
| hsa-miR-200b-5p | SP3 | NM_001172712 | 98.53 | hsa-miR-517a-3p | ZNF521 | NM_015461 | 96.74 |
| hsa-miR-200a-5p | ZC3H12C | NM_033390 | 98.51 | hsa-miR-517b-3p | ZNF521 | NM_015461 | 96.74 |
| hsa-miR-200b-5p | ZC3H12C | NM_033390 | 98.51 | hsa-miR-517c-3p | ZNF521 | NM_015461 | 96.74 |
| hsa-miR-4279 | INHBC | NM_005538 | 98.45 | hsa-miR-4279 | TLK2 | NM_001284333 | 96.66 |
| hsa-miR-8064 | PCGF5 | NM_001256549 | 98.35 | hsa-miR-4279 | TTLL4 | NM_014640 | 96.64 |
| hsa-miR-124-5p | MEF2A | NM_001130926 | 98.11 | hsa-miR-8064 | BNC2 | NM_017637 | 96.60 |
| hsa-miR-124-5p | AZI2 | NM_001271650 | 98.06 | hsa-miR-124-5p | REV3L | NM_001286432 | 96.58 |
| hsa-miR-517a-3p | PHF13 | NM_153812 | 98.02 | hsa-miR-200a-5p | RAB1A | NM_004161 | 96.58 |
| hsa-miR-517b-3p | PHF13 | NM_153812 | 98.02 | hsa-miR-200b-5p | RAB1A | NM_004161 | 96.58 |
| hsa-miR-517c-3p | PHF13 | NM_153812 | 98.02 | hsa-miR-124-5p | PAGR1 | NM_024516 | 96.40 |
| hsa-miR-124-5p | IL6ST | NM_175767 | 97.77 | hsa-miR-642b-5p | ZMYND11 | NM_001202464 | 96.37 |
| hsa-miR-4279 | CACNA1E | NM_001205293 | 97.73 | hsa-miR-4279 | MYRFL | NM_182530 | 96.18 |
| hsa-miR-200a-5p | ATAD2 | NM_014109 | 97.63 | hsa-miR-200a-5p | PPAP2B | NM_003713 | 96.17 |
| hsa-miR-200b-5p | ATAD2 | NM_014109 | 97.63 | hsa-miR-200b-5p | PPAP2B | NM_003713 | 96.17 |
| hsa-miR-200a-5p | SHPRH | NM_173082 | 97.56 | hsa-miR-4279 | GK5 | NM_001039547 | 96.15 |
| hsa-miR-200b-5p | SHPRH | NM_173082 | 97.56 | hsa-miR-124-5p | DPP10 | NM_001321906 | 96.02 |
| hsa-miR-200a-5p | IL1RAP | NM_001167930 | 97.45 | hsa-miR-8064 | VSTM4 | NM_144984 | 95.94 |
| hsa-miR-200b-5p | IL1RAP | NM_001167930 | 97.45 | hsa-miR-4536-3p | PHF13 | NM_153812 | 95.88 |
| hsa-miR-517a-3p | VSTM2B | NM_001146339 | 97.44 | hsa-miR-200b-5p | NDFIP2 | NM_001161407 | 95.73 |
| hsa-miR-517b-3p | VSTM2B | NM_001146339 | 97.44 | hsa-miR-8064 | SF3B1 | NM_001308824 | 95.69 |
| hsa-miR-517c-3p | VSTM2B | NM_001146339 | 97.44 | hsa-miR-200a-5p | UBR3 | NM_172070 | 95.64 |
| hsa-miR-8064 | ELOVL5 | NM_001301856 | 97.29 | hsa-miR-200b-5p | UBR3 | NM_172070 | 95.64 |
| miRNA_v22 | Gene Symbol | miRDB_mRNA | Score | miRNA_v22 | Gene Symbol | miRDB_mRNA | Score |
| hsa-miR-4279 | RHD | NM_001282869 | 95.61 | hsa-miR-4279 | TANC1 | NM_001145909 | 94.00 |
| hsa-miR-4279 | GTF2F2 | NM_004128 | 95.56 | hsa-miR-124-5p | CSTF2 | NM_001306206 | 93.99 |
| hsa-miR-4279 | CAMK1D | NM_153498 | 95.45 | hsa-miR-200a-5p | COL4A1 | NM_001303110 | 93.98 |
| hsa-miR-200a-5p | TMEM39A | NM_018266 | 95.45 | hsa-miR-200b-5p | COL4A1 | NM_001303110 | 93.98 |
| hsa-miR-200b-5p | TMEM39A | NM_018266 | 95.45 | hsa-miR-200a-5p | ZNF440 | NM_152357 | 93.97 |
| hsa-miR-4279 | SOX4 | NM_003107 | 95.18 | hsa-miR-200b-5p | ZNF440 | NM_152357 | 93.97 |
| hsa-miR-8064 | PAFAH1B1 | NM_000430 | 95.13 | hsa-miR-4279 | OAZ1 | NM_001301020 | 93.95 |
| hsa-miR-124-5p | SEC62 | NM_003262 | 94.99 | hsa-miR-124-5p | SPPL3 | NM_139015 | 93.93 |
| hsa-miR-200a-5p | COPS8 | NM_198189 | 94.98 | hsa-miR-200a-5p | EIF4ENIF1 | NM_019843 | 93.89 |
| hsa-miR-200b-5p | COPS8 | NM_198189 | 94.98 | hsa-miR-200b-5p | EIF4ENIF1 | NM_019843 | 93.89 |
| hsa-miR-8064 | DUSP10 | NM_007207 | 94.81 | hsa-miR-4536-3p | ZNF521 | NM_001308225 | 93.88 |
| hsa-miR-200a-5p | SCOC | NM_001153446 | 94.81 | hsa-miR-8064 | ASTN1 | NM_004319 | 93.87 |
| hsa-miR-200b-5p | SCOC | NM_001153446 | 94.81 | hsa-miR-200a-5p | ALX4 | NM_021926 | 93.86 |
| hsa-miR-8064 | UHRF2 | NM_152896 | 94.75 | hsa-miR-200b-5p | ALX4 | NM_021926 | 93.86 |
| hsa-miR-642b-5p | AAK1 | NM_014911 | 94.74 | hsa-miR-4279 | HAPLN1 | NM_001884 | 93.85 |
| hsa-miR-4279 | ROR1 | NM_005012 | 94.73 | hsa-miR-124-5p | TSPAN8 | NM_004616 | 93.82 |
| hsa-miR-642b-5p | SEC24D | NM_001318066 | 94.72 | hsa-miR-642b-5p | ROBO1 | NM_002941 | 93.80 |
| hsa-miR-4279 | INSR | NM_000208 | 94.59 | hsa-miR-4279 | FBXO32 | NM_058229 | 93.75 |
| hsa-miR-8064 | FAM69C | NM_001044369 | 94.49 | hsa-miR-642b-5p | BRINP3 | NM_199051 | 93.72 |
| hsa-miR-124-5p | RBM11 | NM_001320602 | 94.46 | hsa-miR-642b-5p | DYNLL1 | NM_001037494 | 93.66 |
| hsa-miR-200a-5p | NDFIP2 | NM_001161407 | 94.40 | hsa-miR-200a-5p | HMP19 | NM_015980 | 93.62 |
| hsa-miR-4536-3p | VSTM2B | NM_001146339 | 94.39 | hsa-miR-200b-5p | HMP19 | NM_015980 | 93.62 |
| hsa-miR-4279 | KDELC2 | NM_153705 | 94.38 | hsa-miR-8064 | RLF | NM_012421 | 93.59 |
| hsa-miR-124-5p | ERCC6L2 | NM_020207 | 94.30 | hsa-miR-200a-5p | ZNF367 | NM_153695 | 93.50 |
| hsa-miR-124-5p | ACAD10 | NM_025247 | 94.26 | hsa-miR-200b-5p | ZNF367 | NM_153695 | 93.50 |
| hsa-miR-200a-5p | BEST3 | NM_001282616 | 94.22 | hsa-miR-4279 | ZSWIM5 | NM_020883 | 93.50 |
| hsa-miR-200b-5p | BEST3 | NM_001282616 | 94.22 | hsa-miR-4279 | MCHR2 | NM_001040179 | 93.47 |
| hsa-miR-642b-5p | RAP1B | NM_001251922 | 94.18 | hsa-miR-200b-5p | ZNF493 | NM_175910 | 93.45 |
| hsa-miR-642b-5p | KDM3B | NM_016604 | 94.18 | hsa-miR-8064 | DMD | NM_000109 | 93.43 |
| miRNA_v22 | Gene Symbol | miRDB_mRNA | Score | miRNA_v22 | Gene Symbol | miRDB_mRNA | Score |
| hsa-miR-642b-5p | AP1S2 | NM_001272071 | 93.37 | hsa-miR-200a-5p | ZNF763 | NM_001012753 | 92.53 |
| hsa-miR-200a-5p | ATG10 | NM_031482 | 93.37 | hsa-miR-200b-5p | ZNF763 | NM_001012753 | 92.53 |
| hsa-miR-200b-5p | ATG10 | NM_031482 | 93.37 | hsa-miR-4279 | TNRC6B | NM_015088 | 92.51 |
| hsa-miR-4279 | CTDSPL2 | NM_016396 | 93.24 | hsa-miR-200a-5p | TXNRD3 | NM_052883 | 92.48 |
| hsa-miR-200a-5p | USP53 | NM_019050 | 93.21 | hsa-miR-200b-5p | TXNRD3 | NM_052883 | 92.48 |
| hsa-miR-200b-5p | USP53 | NM_019050 | 93.21 | hsa-miR-124-5p | KIAA0430 | NM_014647 | 92.48 |
| hsa-miR-642b-5p | KIF5C | NM_004522 | 93.21 | hsa-miR-124-5p | RSU1 | NM_152724 | 92.44 |
| hsa-miR-517a-3p | HOXA5 | NM_019102 | 93.20 | hsa-miR-4279 | USH2A | NM_206933 | 92.43 |
| hsa-miR-517b-3p | HOXA5 | NM_019102 | 93.20 | hsa-miR-642b-5p | DYNC2H1 | NM_001377 | 92.42 |
| hsa-miR-517c-3p | HOXA5 | NM_019102 | 93.20 | hsa-miR-200a-5p | SGK1 | NM_001143678 | 92.34 |
| hsa-miR-124-5p | DPH6 | NM_001141972 | 93.15 | hsa-miR-200b-5p | SGK1 | NM_001143678 | 92.34 |
| hsa-miR-4279 | PPTC7 | NM_139283 | 93.13 | hsa-miR-200a-5p | TIAM2 | NM_012454 | 92.33 |
| hsa-miR-124-5p | MMADHC | NM_015702 | 93.06 | hsa-miR-200b-5p | TIAM2 | NM_012454 | 92.33 |
| hsa-miR-4279 | CHMP7 | NM_001317899 | 93.02 | hsa-miR-8064 | YOD1 | NM_018566 | 92.26 |
| hsa-miR-642b-5p | ADCY6 | NM_015270 | 93.00 | hsa-miR-124-5p | P4HB | NM_000918 | 92.24 |
| hsa-miR-124-5p | TMEM170B | NM_001100829 | 92.99 | hsa-miR-8064 | ZAK | NM_133646 | 92.22 |
| hsa-miR-4279 | CREBBP | NM_001079846 | 92.97 | hsa-miR-8064 | GADL1 | NM_207359 | 92.19 |
| hsa-miR-4279 | TIPRL | NM_152902 | 92.93 | hsa-miR-4536-3p | DGKH | NM_001204504 | 92.17 |
| hsa-miR-124-5p | ANK3 | NM_020987 | 92.93 | hsa-miR-124-5p | TMEM257 | NM_004709 | 92.17 |
| hsa-miR-642b-5p | PRDX6 | NM_004905 | 92.92 | hsa-miR-4279 | RWDD1 | NM_001007464 | 92.12 |
| hsa-miR-4279 | NRAS | NM_002524 | 92.92 | hsa-miR-8064 | DCUN1D4 | NM_001287757 | 92.11 |
| hsa-miR-124-5p | MGAT4A | NM_012214 | 92.87 | hsa-miR-517a-3p | CBLN2 | NM_182511 | 92.10 |
| hsa-miR-4279 | FGG | NM_021870 | 92.71 | hsa-miR-517b-3p | CBLN2 | NM_182511 | 92.10 |
| hsa-miR-200a-5p | CRLF3 | NM_015986 | 92.69 | hsa-miR-517c-3p | CBLN2 | NM_182511 | 92.10 |
| hsa-miR-200b-5p | CRLF3 | NM_015986 | 92.69 | hsa-miR-4279 | USP18 | NM_017414 | 92.02 |
| hsa-miR-4279 | STAG2 | NM_001042749 | 92.64 | hsa-miR-4279 | EBLN2 | NM_018029 | 91.98 |
| hsa-miR-642b-5p | ORMDL3 | NM_139280 | 92.60 | hsa-miR-642b-5p | SLC35B3 | NM_001142541 | 91.97 |
| hsa-miR-8064 | MAP3K5 | NM_005923 | 92.59 | hsa-miR-4279 | OXR1 | NM_001198534 | 91.93 |
| hsa-miR-124-5p | CLCN4 | NM_001830 | 92.56 | hsa-miR-200a-5p | FOXC1 | NM_001453 | 91.93 |
| miRNA_v22 | Gene Symbol | miRDB_mRNA | Score | miRNA_v22 | Gene Symbol | miRDB_mRNA | Score |
| hsa-miR-200b-5p | FOXC1 | NM_001453 | 91.93 | hsa-miR-642b-5p | RFXANK | NM_001278727 | 90.81 |
| hsa-miR-4279 | ZNF80 | NM_007136 | 91.91 | hsa-miR-4279 | SH3KBP1 | NM_001184960 | 90.80 |
| hsa-miR-8064 | BCL11A | NM_022893 | 91.80 | hsa-miR-4536-3p | ERC2 | NM_015576 | 90.79 |
| hsa-miR-124-5p | CAPRIN1 | NM_005898 | 91.66 | hsa-miR-4279 | YTHDF3 | NM_001277813 | 90.78 |
| hsa-miR-4279 | ATP8A2 | NM_001313741 | 91.66 | hsa-miR-4279 | MSL1 | NM_001012241 | 90.78 |
| hsa-miR-4536-3p | TMEM150C | NM_001080506 | 91.60 | hsa-miR-200a-5p | RANBP3 | NM_003624 | 90.76 |
| hsa-miR-4279 | BCL9 | NM_004326 | 91.57 | hsa-miR-200b-5p | RANBP3 | NM_003624 | 90.76 |
| hsa-miR-642b-5p | ASPH | NM_001164756 | 91.52 | hsa-miR-4279 | TSPAN7 | NM_004615 | 90.72 |
| hsa-miR-124-5p | ARMT1 | NM_024573 | 91.50 | hsa-miR-4279 | DYNC1LI1 | NM_016141 | 90.72 |
| hsa-miR-200a-5p | ZNF396 | NM_145756 | 91.49 | hsa-miR-124-5p | SNX2 | NM_001278199 | 90.70 |
| hsa-miR-200b-5p | ZNF396 | NM_145756 | 91.49 | hsa-miR-200a-5p | ZNF493 | NM_175910 | 90.65 |
| hsa-miR-642b-5p | KCNJ2 | NM_000891 | 91.41 | hsa-miR-4279 | ETV1 | NM_001163149 | 90.58 |
| hsa-miR-8064 | CHIC2 | NM_012110 | 91.35 | hsa-miR-200a-5p | ASNSD1 | NM_019048 | 90.57 |
| hsa-miR-4279 | KCNMA1 | NM_001322838 | 91.32 | hsa-miR-200b-5p | ASNSD1 | NM_019048 | 90.57 |
| hsa-miR-4279 | NDST1 | NM_001543 | 91.31 | hsa-miR-8064 | SPTBN1 | NM_003128 | 90.56 |
| hsa-miR-642b-5p | PROSER1 | NM_025138 | 91.29 | hsa-miR-200a-5p | PPRC1 | NM_001288727 | 90.53 |
| hsa-miR-200a-5p | INSIG2 | NM_001321329 | 91.24 | hsa-miR-200b-5p | PPRC1 | NM_001288727 | 90.53 |
| hsa-miR-200b-5p | INSIG2 | NM_001321329 | 91.24 | hsa-miR-4279 | NMD3 | NM_015938 | 90.51 |
| hsa-miR-642b-5p | JAK2 | NM_001322194 | 91.18 | hsa-miR-4279 | ARHGAP26 | NM_015071 | 90.50 |
| hsa-miR-4279 | SNX19 | NM_001301089 | 91.15 | hsa-miR-642b-5p | PTGER2 | NM_000956 | 90.49 |
| hsa-miR-4279 | KSR2 | NM_173598 | 91.14 | hsa-miR-4279 | KIAA2018 | NM_001009899 | 90.45 |
| hsa-miR-200a-5p | IFT52 | NM_001323579 | 91.03 | hsa-miR-200a-5p | HAUS6 | NM_017645 | 90.45 |
| hsa-miR-200b-5p | IFT52 | NM_001323579 | 91.03 | hsa-miR-200b-5p | HAUS6 | NM_017645 | 90.45 |
| hsa-miR-200a-5p | KIAA1217 | NM_001282770 | 91.03 | hsa-miR-4279 | DGKG | NM_001080744 | 90.41 |
| hsa-miR-200b-5p | KIAA1217 | NM_001282770 | 91.03 | hsa-miR-4279 | SLFN13 | NM_144682 | 90.35 |
| hsa-miR-4279 | SSBP2 | NM_001256734 | 91.03 | hsa-miR-517a-3p | DBN1 | NM_080881 | 90.35 |
| hsa-miR-4279 | DSE | NM_001322944 | 91.00 | hsa-miR-517b-3p | DBN1 | NM_080881 | 90.35 |
| hsa-miR-8064 | TNRC18 | NM_001080495 | 90.97 | hsa-miR-517c-3p | DBN1 | NM_080881 | 90.35 |
| hsa-miR-124-5p | SELENOF | NM_004261 | 90.88 | hsa-miR-4279 | GLI1 | NM_005269 | 90.33 |
| miRNA_v22 | Gene Symbol | miRDB_mRNA | Score | miRNA_v22 | Gene Symbol | miRDB_mRNA | Score |
| hsa-miR-200a-5p | ATP6V0A2 | NM_012463 | 90.32 | hsa-miR-200b-5p | MORC3 | NM_001320445 | 89.41 |
| hsa-miR-200b-5p | ATP6V0A2 | NM_012463 | 90.32 | hsa-miR-8064 | SLC6A19 | NM_001003841 | 89.36 |
| hsa-miR-4279 | TFCP2 | NM_001173452 | 90.26 | hsa-miR-124-5p | ICE1 | NM_015325 | 89.33 |
| hsa-miR-4279 | MPC2 | NM_001143674 | 90.22 | hsa-miR-200a-5p | ZNF91 | NM_001300951 | 89.27 |
| hsa-miR-200a-5p | SNX4 | NM_003794 | 90.19 | hsa-miR-200b-5p | ZNF91 | NM_001300951 | 89.27 |
| hsa-miR-200b-5p | SNX4 | NM_003794 | 90.19 | hsa-miR-8064 | UBE2N | NM_003348 | 89.27 |
| hsa-miR-4536-3p | IGLON5 | NM_001101372 | 90.17 | hsa-miR-4279 | HIPK2 | NM_022740 | 89.26 |
| hsa-miR-124-5p | ZNF117 | NM_015852 | 90.16 | hsa-miR-4279 | TCF21 | NM_003206 | 89.26 |
| hsa-miR-8064 | LPGAT1 | NM_014873 | 90.15 | hsa-miR-4279 | GLCCI1 | NM_138426 | 89.23 |
| hsa-miR-200a-5p | GOLGA7B | NM_001010917 | 90.12 | hsa-miR-642b-5p | YTHDF3 | NM_001277813 | 89.18 |
| hsa-miR-200b-5p | GOLGA7B | NM_001010917 | 90.12 | hsa-miR-124-5p | DNAJB7 | NM_145174 | 89.16 |
| hsa-miR-642b-5p | AKAP1 | NM_001242902 | 90.12 | hsa-miR-642b-5p | NIN | NM_020921 | 89.11 |
| hsa-miR-4279 | ADGRL3 | NM_001322246 | 90.04 | hsa-miR-4279 | C10orf10 | NM_007021 | 89.11 |
| hsa-miR-517a-3p | TMCC1 | NM_001017395 | 89.91 | hsa-miR-4279 | RPTN | NM_001122965 | 89.07 |
| hsa-miR-517b-3p | TMCC1 | NM_001017395 | 89.91 | hsa-miR-4536-3p | FAM179B | NM_001308120 | 89.07 |
| hsa-miR-517c-3p | TMCC1 | NM_001017395 | 89.91 | hsa-miR-8064 | CDK5R1 | NM_003885 | 89.05 |
| hsa-miR-124-5p | CYLC2 | NM_001340 | 89.86 | hsa-miR-200a-5p | SEMA3E | NM_012431 | 89.05 |
| hsa-miR-4279 | ARSJ | NM_024590 | 89.86 | hsa-miR-200b-5p | SEMA3E | NM_012431 | 89.05 |
| hsa-miR-4279 | HDAC6 | NM_001321230 | 89.85 | hsa-miR-200a-5p | HCFC2 | NM_013320 | 89.00 |
| hsa-miR-8064 | FBXO30 | NM_032145 | 89.83 | hsa-miR-200b-5p | HCFC2 | NM_013320 | 89.00 |
| hsa-miR-8064 | DYNC1I1 | NM_001278421 | 89.63 | hsa-miR-4279 | SSR3 | NM_001308205 | 88.96 |
| hsa-miR-642b-5p | LATS1 | NM_004690 | 89.57 | hsa-miR-124-5p | UHRF1 | NM_001048201 | 88.96 |
| hsa-miR-8064 | FRMD4B | NM_015123 | 89.55 | hsa-miR-4279 | EZH1 | NM_001321082 | 88.86 |
| hsa-miR-8064 | GEN1 | NM_001130009 | 89.54 | hsa-miR-642b-5p | GAN | NM_022041 | 88.83 |
| hsa-miR-4279 | SDHC | NM_003001 | 89.54 | hsa-miR-4279 | TTLL2 | NM_031949 | 88.82 |
| hsa-miR-8064 | C2orf49 | NM_001286537 | 89.53 | hsa-miR-4279 | PDF | NM_022341 | 88.81 |
| hsa-miR-200a-5p | HPDL | NM_032756 | 89.42 | hsa-miR-4279 | C11orf87 | NM_207645 | 88.78 |
| hsa-miR-200b-5p | HPDL | NM_032756 | 89.42 | hsa-miR-4279 | PDGFC | NM_016205 | 88.76 |
| hsa-miR-200a-5p | MORC3 | NM_001320445 | 89.41 | hsa-miR-124-5p | RFWD3 | NM_018124 | 88.74 |
| miRNA_v22 | Gene Symbol | miRDB_mRNA | Score | miRNA_v22 | Gene Symbol | miRDB_mRNA | Score |
| hsa-miR-4279 | STXBP5L | NM_014980 | 88.60 | hsa-miR-124-5p | HNRNPF | NM_001098204 | 88.03 |
| hsa-miR-4279 | LAMA1 | NM_005559 | 88.59 | hsa-miR-4279 | FBXO45 | NM_001105573 | 88.01 |
| hsa-miR-4279 | PELI1 | NM_020651 | 88.57 | hsa-miR-642b-5p | RHOBTB3 | NM_014899 | 88.00 |
| hsa-miR-4279 | RALGAPB | NM_001282918 | 88.57 | hsa-miR-124-5p | DDIAS | NM_145018 | 87.99 |
| hsa-miR-4279 | TTYH2 | NM_032646 | 88.56 | hsa-miR-124-5p | DDX6 | NM_001257191 | 87.96 |
| hsa-miR-200a-5p | EIF1AX | NM_001412 | 88.52 | hsa-miR-200a-5p | NDFIP1 | NM_030571 | 87.93 |
| hsa-miR-200b-5p | EIF1AX | NM_001412 | 88.52 | hsa-miR-200b-5p | NDFIP1 | NM_030571 | 87.93 |
| hsa-miR-8064 | SMG7 | NM_001174061 | 88.47 | hsa-miR-200a-5p | ZNF675 | NM_138330 | 87.87 |
| hsa-miR-200a-5p | HOXA10 | NM_018951 | 88.43 | hsa-miR-200b-5p | ZNF675 | NM_138330 | 87.87 |
| hsa-miR-200b-5p | HOXA10 | NM_018951 | 88.43 | hsa-miR-4279 | PCYOX1L | NM_001301054 | 87.87 |
| hsa-miR-642b-5p | ZNF521 | NM_015461 | 88.43 | hsa-miR-8064 | UBR4 | NM_020765 | 87.83 |
| hsa-miR-642b-5p | GABPB2 | NM_001323908 | 88.41 | hsa-miR-4279 | ARID1A | NM_139135 | 87.78 |
| hsa-miR-4279 | CASK | NM_003688 | 88.37 | hsa-miR-642b-5p | ELK4 | NM_001973 | 87.68 |
| hsa-miR-8064 | SMG1 | NM_015092 | 88.32 | hsa-miR-124-5p | IMPA1 | NM_001144879 | 87.64 |
| hsa-miR-200a-5p | NR4A1 | NM_002135 | 88.31 | hsa-miR-200b-5p | HIVEP3 | NM_024503 | 87.62 |
| hsa-miR-200b-5p | NR4A1 | NM_002135 | 88.31 | hsa-miR-200a-5p | MOBP | NM_001278322 | 87.61 |
| hsa-miR-642b-5p | ARHGAP5 | NM_001030055 | 88.28 | hsa-miR-200b-5p | MOBP | NM_001278322 | 87.61 |
| hsa-miR-4279 | SMARCA1 | NM_001282875 | 88.25 | hsa-miR-124-5p | VPS36 | NM_001282168 | 87.61 |
| hsa-miR-124-5p | BORCS7 | NM_001136200 | 88.23 | hsa-miR-4279 | MMD2 | NM_001100600 | 87.58 |
| hsa-miR-4279 | COG8 | NM_032382 | 88.20 | hsa-miR-8064 | C1orf216 | NM_152374 | 87.54 |
| hsa-miR-124-5p | GPC6 | NM_005708 | 88.17 | hsa-miR-124-5p | LRRC28 | NM_001321675 | 87.53 |
| hsa-miR-124-5p | FLRT3 | NM_198391 | 88.11 | hsa-miR-4279 | OLFML2A | NM_001282715 | 87.52 |
| hsa-miR-124-5p | ZNF75A | NM_001324041 | 88.10 | hsa-miR-124-5p | MMGT1 | NM_173470 | 87.47 |
| hsa-miR-517a-3p | PTK2B | NM_004103 | 88.10 | hsa-miR-4279 | STON2 | NM_033104 | 87.40 |
| hsa-miR-517b-3p | PTK2B | NM_004103 | 88.10 | hsa-miR-124-5p | NOA1 | NM_032313 | 87.36 |
| hsa-miR-517c-3p | PTK2B | NM_004103 | 88.10 | hsa-miR-517a-3p | NFIC | NM_001245005 | 87.33 |
| hsa-miR-124-5p | MTDH | NM_178812 | 88.09 | hsa-miR-517b-3p | NFIC | NM_001245005 | 87.33 |
| hsa-miR-642b-5p | WDR45B | NM_019613 | 88.09 | hsa-miR-517c-3p | NFIC | NM_001245005 | 87.33 |
| hsa-miR-8064 | AAK1 | NM_014911 | 88.04 | hsa-miR-642b-5p | PAPD5 | NM_001040284 | 87.32 |
| miRNA_v22 | Gene Symbol | miRDB_mRNA | Score | miRNA_v22 | Gene Symbol | miRDB_mRNA | Score |
| hsa-miR-8064 | ITGAM | NM_000632 | 87.29 | hsa-miR-517a-3p | CCDC89 | NM_152723 | 86.83 |
| hsa-miR-200b-5p | GRHL1 | NM_198182 | 87.25 | hsa-miR-517b-3p | CCDC89 | NM_152723 | 86.83 |
| hsa-miR-517a-3p | SMIM14 | NM_001317896 | 87.25 | hsa-miR-517c-3p | CCDC89 | NM_152723 | 86.83 |
| hsa-miR-517b-3p | SMIM14 | NM_001317896 | 87.25 | hsa-miR-200a-5p | SERINC3 | NM_006811 | 86.80 |
| hsa-miR-517c-3p | SMIM14 | NM_001317896 | 87.25 | hsa-miR-200b-5p | SERINC3 | NM_006811 | 86.80 |
| hsa-miR-200a-5p | RAB11B | NM_004218 | 87.24 | hsa-miR-4279 | STK35 | NM_080836 | 86.80 |
| hsa-miR-200b-5p | RAB11B | NM_004218 | 87.24 | hsa-miR-4279 | ARHGEF28 | NM_001177693 | 86.79 |
| hsa-miR-4279 | KIAA1958 | NM_001287038 | 87.23 | hsa-miR-4279 | TVP23C | NM_145301 | 86.78 |
| hsa-miR-4536-3p | DBN1 | NM_004395 | 87.20 | hsa-miR-200a-5p | C11orf53 | NM_198498 | 86.74 |
| hsa-miR-4279 | AFF1 | NM_001313960 | 87.18 | hsa-miR-200b-5p | C11orf53 | NM_198498 | 86.74 |
| hsa-miR-4279 | MGAT3 | NM_001098270 | 87.17 | hsa-miR-124-5p | HNRNPH1 | NM_001257293 | 86.69 |
| hsa-miR-4279 | PLXNA2 | NM_025179 | 87.15 | hsa-miR-642b-5p | CTNNB1 | NM_001904 | 86.67 |
| hsa-miR-642b-5p | CALM2 | NM_001305624 | 87.11 | hsa-miR-4279 | CLCN3 | NM_001243372 | 86.63 |
| hsa-miR-4279 | SLC1A2 | NM_004171 | 87.09 | hsa-miR-124-5p | PPP2R5E | NM_001282180 | 86.63 |
| hsa-miR-4279 | MARCHF1 | NM_001166373 | 87.07 | hsa-miR-200a-5p | THAP5 | NM_001287601 | 86.61 |
| hsa-miR-4536-3p | NFIA | NM_001134673 | 87.04 | hsa-miR-200b-5p | THAP5 | NM_001287601 | 86.61 |
| hsa-miR-517a-3p | NFIB | NM_001190737 | 87.04 | hsa-miR-8064 | C7orf73 | NM_001130929 | 86.60 |
| hsa-miR-517b-3p | NFIB | NM_001190737 | 87.04 | hsa-miR-124-5p | CELF2 | NM_001025076 | 86.53 |
| hsa-miR-517c-3p | NFIB | NM_001190737 | 87.04 | hsa-miR-124-5p | ZNF266 | NM_006631 | 86.53 |
| hsa-miR-4279 | TMEM65 | NM_194291 | 87.01 | hsa-miR-200a-5p | FAM208B | NM_001321783 | 86.52 |
| hsa-miR-8064 | ADAM17 | NM_003183 | 87.00 | hsa-miR-200b-5p | FAM208B | NM_001321783 | 86.52 |
| hsa-miR-8064 | KCNMA1 | NM_001322837 | 86.97 | hsa-miR-8064 | PPP1CB | NM_002709 | 86.49 |
| hsa-miR-124-5p | ATP1B1 | NM_001677 | 86.94 | hsa-miR-124-5p | STRBP | NM_001171137 | 86.49 |
| hsa-miR-642b-5p | PRKRA | NM_003690 | 86.90 | hsa-miR-124-5p | DCLK1 | NM_001195415 | 86.47 |
| hsa-miR-642b-5p | SP100 | NM_001080391 | 86.90 | hsa-miR-4536-3p | SRSF1 | NM_001078166 | 86.46 |
| hsa-miR-124-5p | ZNF782 | NM_001001662 | 86.88 | hsa-miR-4279 | THY1 | NM_001311162 | 86.45 |
| hsa-miR-4279 | ATXN1 | NM_000332 | 86.86 | hsa-miR-8064 | AGO3 | NM_024852 | 86.44 |
| hsa-miR-642b-5p | HAPLN1 | NM_001884 | 86.86 | hsa-miR-642b-5p | EIF1 | NM_005801 | 86.39 |
| hsa-miR-642b-5p | LDHA | NM_001135239 | 86.85 | hsa-miR-200a-5p | ARL13B | NM_001321328 | 86.36 |
| miRNA_v22 | Gene Symbol | miRDB_mRNA | Score | miRNA_v22 | Gene Symbol | miRDB_mRNA | Score |
| hsa-miR-200b-5p | ARL13B | NM_001321328 | 86.36 | hsa-miR-124-5p | DUSP19 | NM_001321519 | 85.95 |
| hsa-miR-4279 | TMX3 | NM_019022 | 86.36 | hsa-miR-124-5p | ERICH1 | NM_001303100 | 85.95 |
| hsa-miR-8064 | AGBL3 | NM_178563 | 86.35 | hsa-miR-200a-5p | NFIA | NM_001145512 | 85.94 |
| hsa-miR-4279 | STXBP5 | NM_001127715 | 86.33 | hsa-miR-200b-5p | NFIA | NM_001145512 | 85.94 |
| hsa-miR-8064 | DOK3 | NM_001308236 | 86.32 | hsa-miR-200a-5p | SLC25A40 | NM_018843 | 85.85 |
| hsa-miR-4279 | ANKS6 | NM_173551 | 86.26 | hsa-miR-200b-5p | SLC25A40 | NM_018843 | 85.85 |
| hsa-miR-642b-5p | ATXN7L3 | NM_020218 | 86.26 | hsa-miR-4279 | KCNB1 | NM_004975 | 85.84 |
| hsa-miR-124-5p | TFAP2A | NM_001042425 | 86.26 | hsa-miR-642b-5p | ZNF544 | NM_001320789 | 85.84 |
| hsa-miR-124-5p | ASB15 | NM_080928 | 86.25 | hsa-miR-200a-5p | PPM1E | NM_014906 | 85.83 |
| hsa-miR-8064 | PPP3CA | NM_000944 | 86.25 | hsa-miR-200b-5p | PPM1E | NM_014906 | 85.83 |
| hsa-miR-4279 | CLASP2 | NM_015097 | 86.22 | hsa-miR-8064 | VDAC2 | NM_001184823 | 85.82 |
| hsa-miR-4536-3p | LEMD3 | NM_014319 | 86.21 | hsa-miR-124-5p | FAR1 | NM_032228 | 85.80 |
| hsa-miR-124-5p | SPIN1 | NM_006717 | 86.19 | hsa-miR-642b-5p | ANKMY2 | NM_020319 | 85.80 |
| hsa-miR-200a-5p | KMT2E | NM_182931 | 86.18 | hsa-miR-4279 | GFPT2 | NM_005110 | 85.79 |
| hsa-miR-200b-5p | KMT2E | NM_182931 | 86.18 | hsa-miR-124-5p | ZBTB20 | NM_001164343 | 85.75 |
| hsa-miR-642b-5p | SLC30A9 | NM_006345 | 86.13 | hsa-miR-124-5p | TAB2 | NM_001292034 | 85.74 |
| hsa-miR-4279 | KIAA2022 | NM_001008537 | 86.13 | hsa-miR-4279 | SNX27 | NM_030918 | 85.73 |
| hsa-miR-124-5p | RIC3 | NM_024557 | 86.13 | hsa-miR-200a-5p | PKP2 | NM_004572 | 85.72 |
| hsa-miR-4279 | ERICH5 | NM_001170806 | 86.12 | hsa-miR-200a-5p | ZNF208 | NM_007153 | 85.69 |
| hsa-miR-124-5p | HECTD2 | NM_001284274 | 86.10 | hsa-miR-200b-5p | ZNF208 | NM_007153 | 85.69 |
| hsa-miR-4279 | ADCY6 | NM_015270 | 86.03 | hsa-miR-4536-3p | FOXJ3 | NM_001198850 | 85.68 |
| hsa-miR-124-5p | TXNRD1 | NM_182742 | 86.03 | hsa-miR-642b-5p | GPATCH2L | NM_001322031 | 85.68 |
| hsa-miR-4279 | DBT | NM_001918 | 86.00 | hsa-miR-4536-3p | LRRTM3 | NM_178011 | 85.67 |
| hsa-miR-517a-3p | DGKH | NM_001204504 | 86.00 | hsa-miR-4279 | PWWP2A | NM_052927 | 85.66 |
| hsa-miR-517b-3p | DGKH | NM_001204504 | 86.00 | hsa-miR-8064 | ZNF248 | NM_001267605 | 85.65 |
| hsa-miR-517c-3p | DGKH | NM_001204504 | 86.00 | hsa-miR-4279 | DLX4 | NM_001934 | 85.58 |
| hsa-miR-4279 | CD274 | NM_001267706 | 85.98 | hsa-miR-8064 | PCDH10 | NM_032961 | 85.58 |
| hsa-miR-642b-5p | SLC12A5 | NM_001134771 | 85.97 | hsa-miR-124-5p | TRAF3 | NM_001199427 | 85.55 |
| hsa-miR-124-5p | NR1I2 | NM_003889 | 85.97 | hsa-miR-642b-5p | SERINC3 | NM_006811 | 85.54 |
| miRNA_v22 | Gene Symbol | miRDB_mRNA | Score | miRNA_v22 | Gene Symbol | miRDB_mRNA | Score |
| hsa-miR-4279 | PRICKLE2 | NM_198859 | 85.53 | hsa-miR-642b-5p | RREB1 | NM_001003699 | 85.11 |
| hsa-miR-517a-3p | TNIP1 | NM_001252391 | 85.52 | hsa-miR-200a-5p | AHR | NM_001621 | 85.05 |
| hsa-miR-517b-3p | TNIP1 | NM_001252391 | 85.52 | hsa-miR-200b-5p | AHR | NM_001621 | 85.05 |
| hsa-miR-517c-3p | TNIP1 | NM_001252391 | 85.52 | hsa-miR-4279 | NR6A1 | NM_001489 | 85.01 |
| hsa-miR-124-5p | ZNF326 | NM_181781 | 85.51 | hsa-miR-4279 | TARS2 | NM_025150 | 85.00 |
| hsa-miR-124-5p | MRPS33 | NM_016071 | 85.50 | hsa-miR-642b-5p | TRA2B | NM_001243879 | 84.98 |
| hsa-miR-4279 | SELL | NM_000655 | 85.47 | hsa-miR-200a-5p | LMTK2 | NM_014916 | 84.92 |
| hsa-miR-642b-5p | SIRT1 | NM_001142498 | 85.47 | hsa-miR-200b-5p | LMTK2 | NM_014916 | 84.92 |
| hsa-miR-8064 | GPR161 | NM_001267613 | 85.46 | hsa-miR-4279 | BMF | NM_033503 | 84.84 |
| hsa-miR-642b-5p | ABL2 | NM_005158 | 85.41 | hsa-miR-124-5p | MAPK14 | NM_139014 | 84.82 |
| hsa-miR-4279 | GEMIN5 | NM_001252156 | 85.39 | hsa-miR-124-5p | KCTD15 | NM_024076 | 84.81 |
| hsa-miR-4279 | TBC1D32 | NM_152730 | 85.38 | hsa-miR-4279 | TIFA | NM_052864 | 84.74 |
| hsa-miR-4279 | KCNH5 | NM_172375 | 85.36 | hsa-miR-124-5p | VPS52 | NM_001289174 | 84.72 |
| hsa-miR-4279 | CLOCK | NM_004898 | 85.36 | hsa-miR-200a-5p | WHSC1 | NM_133334 | 84.66 |
| hsa-miR-124-5p | FOXG1 | NM_005249 | 85.34 | hsa-miR-200b-5p | WHSC1 | NM_133334 | 84.66 |
| hsa-miR-124-5p | SEC24D | NM_001318066 | 85.32 | hsa-miR-4279 | KLF12 | NM_007249 | 84.66 |
| hsa-miR-124-5p | CHTF8 | NM_001039690 | 85.30 | hsa-miR-4279 | KCNN3 | NM_170782 | 84.63 |
| hsa-miR-124-5p | TRIP4 | NM_001321924 | 85.29 | hsa-miR-200a-5p | EPM2AIP1 | NM_014805 | 84.53 |
| hsa-miR-4536-3p | HS3ST3A1 | NM_006042 | 85.29 | hsa-miR-200b-5p | EPM2AIP1 | NM_014805 | 84.53 |
| hsa-miR-200a-5p | KDM2B | NM_032590 | 85.28 | hsa-miR-4279 | GDAP2 | NM_017686 | 84.51 |
| hsa-miR-200b-5p | KDM2B | NM_032590 | 85.28 | hsa-miR-200a-5p | CRIM1 | NM_016441 | 84.49 |
| hsa-miR-4279 | FAM168A | NM_001286050 | 85.26 | hsa-miR-200b-5p | CRIM1 | NM_016441 | 84.49 |
| hsa-miR-642b-5p | MTX3 | NM_001167741 | 85.26 | hsa-miR-4279 | EMCN | NM_001159694 | 84.49 |
| hsa-miR-124-5p | USP6NL | NM_014688 | 85.26 | hsa-miR-4279 | KPNB1 | NM_002265 | 84.44 |
| hsa-miR-642b-5p | NPTX1 | NM_002522 | 85.25 | hsa-miR-642b-5p | PHACTR3 | NM_001199505 | 84.43 |
| hsa-miR-642b-5p | AP3M1 | NM_001320263 | 85.25 | hsa-miR-200a-5p | BBX | NM_020235 | 84.38 |
| hsa-miR-4279 | UBXN4 | NM_014607 | 85.20 | hsa-miR-200b-5p | BBX | NM_020235 | 84.38 |
| hsa-miR-642b-5p | ALPL | NM_001127501 | 85.17 | hsa-miR-4279 | ANKRD13A | NM_033121 | 84.35 |
| hsa-miR-8064 | ATP6AP2 | NM_005765 | 85.16 | hsa-miR-4279 | MARCHF5 | NM_017824 | 84.33 |
| miRNA_v22 | Gene Symbol | miRDB_mRNA | Score | miRNA_v22 | Gene Symbol | miRDB_mRNA | Score |
| hsa-miR-124-5p | LIN7C | NM_018362 | 84.33 | hsa-miR-4279 | GPR162 | NM_019858 | 83.80 |
| hsa-miR-124-5p | KIAA1804 | NM_032435 | 84.32 | hsa-miR-4279 | TSPYL1 | NM_003309 | 83.79 |
| hsa-miR-4279 | ITIH5 | NM_030569 | 84.29 | hsa-miR-4279 | CLIC5 | NM_016929 | 83.79 |
| hsa-miR-4279 | ALG10B | NM_001013620 | 84.29 | hsa-miR-4279 | CYHR1 | NM_138496 | 83.78 |
| hsa-miR-4279 | KMT2C | NM_170606 | 84.29 | hsa-miR-4279 | FOS | NM_005252 | 83.77 |
| hsa-miR-642b-5p | JAG1 | NM_000214 | 84.29 | hsa-miR-8064 | NNAT | NM_005386 | 83.75 |
| hsa-miR-124-5p | SGCZ | NM_001322879 | 84.29 | hsa-miR-124-5p | NSF | NM_006178 | 83.71 |
| hsa-miR-200a-5p | DNAJC27 | NM_016544 | 84.25 | hsa-miR-124-5p | ADAMTS19 | NM_133638 | 83.71 |
| hsa-miR-200b-5p | DNAJC27 | NM_016544 | 84.25 | hsa-miR-4279 | KLHL15 | NM_030624 | 83.70 |
| hsa-miR-4279 | NPR1 | NM_000906 | 84.23 | hsa-miR-4536-3p | ARCN1 | NM_001142281 | 83.68 |
| hsa-miR-642b-5p | PPM1K | NM_152542 | 84.23 | hsa-miR-517a-3p | FOXJ3 | NM_001198850 | 83.61 |
| hsa-miR-4279 | ZNF75A | NM_001324085 | 84.21 | hsa-miR-517b-3p | FOXJ3 | NM_001198850 | 83.61 |
| hsa-miR-4279 | DOK6 | NM_152721 | 84.21 | hsa-miR-517c-3p | FOXJ3 | NM_001198850 | 83.61 |
| hsa-miR-124-5p | SLC35A3 | NM_001271684 | 84.20 | hsa-miR-4279 | CCDC108 | NM_152389 | 83.59 |
| hsa-miR-642b-5p | MCEE | NM_032601 | 84.14 | hsa-miR-200a-5p | KCTD12 | NM_138444 | 83.57 |
| hsa-miR-8064 | ADCYAP1 | NM_001099733 | 84.12 | hsa-miR-200b-5p | KCTD12 | NM_138444 | 83.57 |
| hsa-miR-4279 | KLF3 | NM_016531 | 84.09 | hsa-miR-4279 | MVB12B | NM_001011703 | 83.57 |
| hsa-miR-200a-5p | AMMECR1 | NM_015365 | 84.08 | hsa-miR-8064 | TMEM179 | NM_001286389 | 83.56 |
| hsa-miR-200b-5p | AMMECR1 | NM_015365 | 84.08 | hsa-miR-642b-5p | ITPR1 | NM_001168272 | 83.56 |
| hsa-miR-4279 | GSTCD | NM_001031720 | 84.08 | hsa-miR-4279 | ANKRD6 | NM_001242809 | 83.55 |
| hsa-miR-8064 | CNEP1R1 | NM_001281789 | 84.07 | hsa-miR-517a-3p | SHANK1 | NM_016148 | 83.50 |
| hsa-miR-200a-5p | KLF7 | NM_001270944 | 84.03 | hsa-miR-517b-3p | SHANK1 | NM_016148 | 83.50 |
| hsa-miR-200b-5p | KLF7 | NM_001270944 | 84.03 | hsa-miR-517c-3p | SHANK1 | NM_016148 | 83.50 |
| hsa-miR-4279 | PRDM1 | NM_001198 | 84.02 | hsa-miR-4279 | ZBTB43 | NM_014007 | 83.50 |
| hsa-miR-200a-5p | SLC22A15 | NM_018420 | 84.02 | hsa-miR-200a-5p | KLHL5 | NM_001171654 | 83.41 |
| hsa-miR-200b-5p | SLC22A15 | NM_018420 | 84.02 | hsa-miR-200b-5p | KLHL5 | NM_001171654 | 83.41 |
| hsa-miR-642b-5p | DACH1 | NM_004392 | 83.84 | hsa-miR-642b-5p | FAM155A | NM_001080396 | 83.39 |
| hsa-miR-4279 | PGAM4 | NM_001029891 | 83.81 | hsa-miR-200a-5p | FOXD1 | NM_004472 | 83.37 |
| hsa-miR-8064 | ZBTB33 | NM_001184742 | 83.81 | hsa-miR-200b-5p | FOXD1 | NM_004472 | 83.37 |
| miRNA_v22 | Gene Symbol | miRDB_mRNA | Score | miRNA_v22 | Gene Symbol | miRDB_mRNA | Score |
| hsa-miR-8064 | FILIP1L | NM_001042459 | 83.37 | hsa-miR-124-5p | ZEB2 | NM_001171653 | 82.95 |
| hsa-miR-200a-5p | NCF2 | NM_001127651 | 83.36 | hsa-miR-200a-5p | LRRC57 | NM_153260 | 82.88 |
| hsa-miR-200b-5p | NCF2 | NM_001127651 | 83.36 | hsa-miR-200b-5p | LRRC57 | NM_153260 | 82.88 |
| hsa-miR-8064 | SPRY3 | NM_001304990 | 83.35 | hsa-miR-124-5p | CPEB2 | NM_001177381 | 82.88 |
| hsa-miR-4279 | LYPD6 | NM_001195685 | 83.32 | hsa-miR-642b-5p | LIN28B | NM_001004317 | 82.87 |
| hsa-miR-4279 | STK3 | NM_006281 | 83.31 | hsa-miR-200b-5p | ADRBK2 | NM_005160 | 82.86 |
| hsa-miR-642b-5p | PRAMEF18 | NM_001099850 | 83.30 | hsa-miR-4279 | SNX8 | NM_013321 | 82.86 |
| hsa-miR-4279 | ZNF202 | NM_001301780 | 83.24 | hsa-miR-642b-5p | RBM27 | NM_018989 | 82.85 |
| hsa-miR-200a-5p | AGPAT5 | NM_018361 | 83.24 | hsa-miR-124-5p | TTC14 | NM_133462 | 82.85 |
| hsa-miR-200b-5p | AGPAT5 | NM_018361 | 83.24 | hsa-miR-4279 | BCOR | NM_017745 | 82.85 |
| hsa-miR-200a-5p | SH3RF1 | NM_020870 | 83.23 | hsa-miR-4279 | C20orf202 | NM_001009612 | 82.83 |
| hsa-miR-200b-5p | SH3RF1 | NM_020870 | 83.23 | hsa-miR-642b-5p | GRIA4 | NM_000829 | 82.78 |
| hsa-miR-8064 | CHODL | NM_001204178 | 83.23 | hsa-miR-8064 | A1CF | NM_138932 | 82.78 |
| hsa-miR-4279 | ZNF33A | NM_001324176 | 83.22 | hsa-miR-4279 | MKRN1 | NM_001145125 | 82.77 |
| hsa-miR-8064 | WSB2 | NM_001278557 | 83.19 | hsa-miR-4279 | AMER2 | NM_152704 | 82.74 |
| hsa-miR-4279 | KPNA6 | NM_012316 | 83.15 | hsa-miR-200a-5p | MDFIC | NM_001166345 | 82.73 |
| hsa-miR-4536-3p | NUPL2 | NM_007342 | 83.11 | hsa-miR-200b-5p | MDFIC | NM_001166345 | 82.73 |
| hsa-miR-4279 | COL6A5 | NM_001278298 | 83.09 | hsa-miR-4279 | HECA | NM_016217 | 82.72 |
| hsa-miR-4279 | ANGPT4 | NM_015985 | 83.06 | hsa-miR-4279 | SMPD3 | NM_018667 | 82.72 |
| hsa-miR-517a-3p | CLIC4 | NM_013943 | 83.03 | hsa-miR-4279 | P2RY10 | NM_198333 | 82.72 |
| hsa-miR-517b-3p | CLIC4 | NM_013943 | 83.03 | hsa-miR-200a-5p | OPRM1 | NM_001285526 | 82.70 |
| hsa-miR-517c-3p | CLIC4 | NM_013943 | 83.03 | hsa-miR-200b-5p | OPRM1 | NM_001285526 | 82.70 |
| hsa-miR-124-5p | HNMT | NM_006895 | 83.03 | hsa-miR-4536-3p | OLFM3 | NM_001288823 | 82.63 |
| hsa-miR-642b-5p | BBIP1 | NM_001195304 | 83.02 | hsa-miR-8064 | MYT1L | NM_015025 | 82.61 |
| hsa-miR-4536-3p | IRF2BPL | NM_024496 | 83.01 | hsa-miR-8064 | CRY2 | NM_001127457 | 82.60 |
| hsa-miR-124-5p | SLITRK5 | NM_015567 | 83.00 | hsa-miR-8064 | AQP2 | NM_000486 | 82.58 |
| hsa-miR-4279 | BAZ1A | NM_013448 | 83.00 | hsa-miR-8064 | KCNC2 | NM_001260497 | 82.57 |
| hsa-miR-4279 | PRKAA2 | NM_006252 | 82.98 | hsa-miR-124-5p | TMEM38B | NM_018112 | 82.57 |
| hsa-miR-124-5p | MTERF3 | NM_001286643 | 82.96 | hsa-miR-200a-5p | PCDHB6 | NM_001303145 | 82.56 |
| miRNA_v22 | Gene Symbol | miRDB_mRNA | Score | miRNA_v22 | Gene Symbol | miRDB_mRNA | Score |
| hsa-miR-200b-5p | PCDHB6 | NM_001303145 | 82.56 | hsa-miR-200a-5p | ETFDH | NM_001281738 | 82.13 |
| hsa-miR-4279 | SH3BGRL | NM_003022 | 82.52 | hsa-miR-200b-5p | ETFDH | NM_001281738 | 82.13 |
| hsa-miR-4536-3p | CBLN2 | NM_182511 | 82.52 | hsa-miR-200a-5p | ENKUR | NM_001270383 | 82.11 |
| hsa-miR-4279 | DNAJB4 | NM_001317103 | 82.50 | hsa-miR-200b-5p | ENKUR | NM_001270383 | 82.11 |
| hsa-miR-200a-5p | ZNF254 | NM_001278664 | 82.48 | hsa-miR-8064 | USH1G | NM_173477 | 82.10 |
| hsa-miR-200b-5p | ZNF254 | NM_001278664 | 82.48 | hsa-miR-4279 | XPR1 | NM_001135669 | 82.09 |
| hsa-miR-124-5p | NABP1 | NM_001031716 | 82.47 | hsa-miR-124-5p | VCL | NM_003373 | 82.09 |
| hsa-miR-124-5p | FAM171B | NM_177454 | 82.46 | hsa-miR-8064 | PMAIP1 | NM_021127 | 82.06 |
| hsa-miR-4279 | PAFAH1B2 | NM_002572 | 82.43 | hsa-miR-8064 | LACE1 | NM_001323005 | 82.05 |
| hsa-miR-200a-5p | LIMCH1 | NM_001112717 | 82.40 | hsa-miR-200a-5p | GLTSCR1 | NM_015711 | 82.05 |
| hsa-miR-200b-5p | LIMCH1 | NM_001112717 | 82.40 | hsa-miR-200b-5p | GLTSCR1 | NM_015711 | 82.05 |
| hsa-miR-200b-5p | PKP2 | NM_004572 | 82.38 | hsa-miR-4279 | CPEB4 | NM_001308189 | 82.04 |
| hsa-miR-4279 | ZNF551 | NM_001270938 | 82.36 | hsa-miR-517a-3p | NFIA | NM_005595 | 82.01 |
| hsa-miR-124-5p | CD24 | NM_001291739 | 82.31 | hsa-miR-517b-3p | NFIA | NM_005595 | 82.01 |
| hsa-miR-124-5p | MAP4 | NM_030885 | 82.30 | hsa-miR-517c-3p | NFIA | NM_005595 | 82.01 |
| hsa-miR-4279 | ATAD1 | NM_032810 | 82.29 | hsa-miR-4279 | C1orf56 | NM_017860 | 82.00 |
| hsa-miR-4279 | ATMIN | NM_001300728 | 82.26 | hsa-miR-4279 | KDM2A | NM_012308 | 81.94 |
| hsa-miR-200a-5p | GRHL1 | NM_198182 | 82.26 | hsa-miR-200a-5p | CHD7 | NM_017780 | 81.92 |
| hsa-miR-4279 | GANAB | NM_001278194 | 82.25 | hsa-miR-200b-5p | CHD7 | NM_017780 | 81.92 |
| hsa-miR-124-5p | SGCD | NM_001128209 | 82.25 | hsa-miR-8064 | MAP1B | NM_001324255 | 81.91 |
| hsa-miR-200a-5p | GLS | NM_014905 | 82.23 | hsa-miR-4536-3p | MARCHF9 | NM_138396 | 81.87 |
| hsa-miR-4279 | STXBP3 | NM_007269 | 82.23 | hsa-miR-4279 | THEG5 | NM_001278577 | 81.86 |
| hsa-miR-200a-5p | MIER3 | NM_001297599 | 82.21 | hsa-miR-200a-5p | FGF13 | NM_033642 | 81.86 |
| hsa-miR-200b-5p | MIER3 | NM_001297599 | 82.21 | hsa-miR-200b-5p | FGF13 | NM_033642 | 81.86 |
| hsa-miR-4279 | SIRT5 | NM_001193267 | 82.18 | hsa-miR-124-5p | SLC30A7 | NM_001144884 | 81.85 |
| hsa-miR-200a-5p | SULT1C2 | NM_001056 | 82.18 | hsa-miR-124-5p | LDLRAD4 | NM_001003675 | 81.84 |
| hsa-miR-200b-5p | SULT1C2 | NM_001056 | 82.18 | hsa-miR-4279 | GSTZ1 | NM_145870 | 81.83 |
| hsa-miR-642b-5p | ZNF800 | NM_176814 | 82.15 | hsa-miR-8064 | PNPLA3 | NM_025225 | 81.79 |
| hsa-miR-4279 | DYRK2 | NM_006482 | 82.14 | hsa-miR-8064 | CLIC5 | NM_001114086 | 81.79 |
| miRNA_v22 | Gene Symbol | miRDB_mRNA | Score | miRNA_v22 | Gene Symbol | miRDB_mRNA | Score |
| hsa-miR-124-5p | ARMC1 | NM_001286702 | 81.78 | hsa-miR-4279 | GREM1 | NM_013372 | 80.99 |
| hsa-miR-4279 | STON1-GTF2A1L | NM_001198593 | 81.66 | hsa-miR-124-5p | AFF3 | NM_002285 | 80.96 |
| hsa-miR-642b-5p | TNS1 | NM_001308023 | 81.65 | hsa-miR-4279 | EPAS1 | NM_001430 | 80.95 |
| hsa-miR-4279 | CTXN3 | NM_001127385 | 81.65 | hsa-miR-124-5p | CHKA | NM_212469 | 80.94 |
| hsa-miR-124-5p | FAM208A | NM_015224 | 81.63 | hsa-miR-124-5p | ASH2L | NM_001261832 | 80.93 |
| hsa-miR-200a-5p | TXNDC17 | NM_032731 | 81.51 | hsa-miR-4279 | PELI2 | NM_021255 | 80.91 |
| hsa-miR-200b-5p | TXNDC17 | NM_032731 | 81.51 | hsa-miR-124-5p | USP31 | NM_020718 | 80.90 |
| hsa-miR-4279 | CNOT4 | NM_001190850 | 81.49 | hsa-miR-8064 | UBE2W | NM_018299 | 80.89 |
| hsa-miR-124-5p | MKX | NM_173576 | 81.48 | hsa-miR-4279 | THSD4 | NM_001286429 | 80.88 |
| hsa-miR-124-5p | SEC23A | NM_006364 | 81.48 | hsa-miR-4279 | DCAF7 | NM_005828 | 80.88 |
| hsa-miR-200a-5p | ARC | NM_015193 | 81.47 | hsa-miR-8064 | ANOS1 | NM_000216 | 80.87 |
| hsa-miR-200b-5p | ARC | NM_015193 | 81.47 | hsa-miR-200a-5p | C18orf54 | NM_001288982 | 80.85 |
| hsa-miR-4279 | ZNF426 | NM_001318056 | 81.45 | hsa-miR-200b-5p | C18orf54 | NM_001288982 | 80.85 |
| hsa-miR-4279 | ZNF793 | NM_001013659 | 81.43 | hsa-miR-4536-3p | NR4A3 | NM_006981 | 80.84 |
| hsa-miR-4279 | CENPF | NM_016343 | 81.42 | hsa-miR-4279 | FAM234B | NM_020853 | 80.80 |
| hsa-miR-642b-5p | KLF3 | NM_016531 | 81.39 | hsa-miR-642b-5p | PCTP | NM_021213 | 80.80 |
| hsa-miR-4279 | SLC25A20 | NM_000387 | 81.33 | hsa-miR-124-5p | RAB3IP | NM_175624 | 80.75 |
| hsa-miR-642b-5p | PDZD8 | NM_173791 | 81.26 | hsa-miR-4279 | EMP2 | NM_001424 | 80.75 |
| hsa-miR-8064 | JMY | NM_152405 | 81.20 | hsa-miR-200a-5p | HIVEP3 | NM_024503 | 80.71 |
| hsa-miR-200a-5p | EID1 | NM_014335 | 81.20 | hsa-miR-4279 | CDK6 | NM_001259 | 80.71 |
| hsa-miR-200b-5p | EID1 | NM_014335 | 81.20 | hsa-miR-517a-3p | HERPUD2 | NM_022373 | 80.69 |
| hsa-miR-8064 | SLC6A17 | NM_001010898 | 81.12 | hsa-miR-517b-3p | HERPUD2 | NM_022373 | 80.69 |
| hsa-miR-4279 | GNAL | NM_001261444 | 81.11 | hsa-miR-517c-3p | HERPUD2 | NM_022373 | 80.69 |
| hsa-miR-8064 | ARHGAP32 | NM_001142685 | 81.11 | hsa-miR-4279 | ADORA1 | NM_000674 | 80.64 |
| hsa-miR-8064 | RFX3 | NM_134428 | 81.09 | hsa-miR-4279 | DHRS12 | NM_024705 | 80.62 |
| hsa-miR-124-5p | TSGA10 | NM_025244 | 81.08 | hsa-miR-200a-5p | ZNF138 | NM_001271649 | 80.56 |
| hsa-miR-642b-5p | DNAH9 | NM_004662 | 81.04 | hsa-miR-200b-5p | ZNF138 | NM_001271649 | 80.56 |
| hsa-miR-124-5p | LSM14A | NM_015578 | 81.04 | hsa-miR-124-5p | UVRAG | NM_003369 | 80.54 |
| hsa-miR-124-5p | ANKRD6 | NM_001242811 | 81.01 | hsa-miR-4279 | KCNK10 | NM_138318 | 80.52 |
| miRNA_v22 | Gene Symbol | miRDB_mRNA | Score | miRNA_v22 | Gene Symbol | miRDB_mRNA | Score |
| hsa-miR-8064 | SPAST | NM_199436 | 80.48 | hsa-miR-4279 | PXN | NM_025157 | 80.24 |
| hsa-miR-4279 | WBP2NL | NM_152613 | 80.47 | hsa-miR-4279 | OPA3 | NM_025136 | 80.21 |
| hsa-miR-4279 | CLCN5 | NM_000084 | 80.46 | hsa-miR-642b-5p | DMTF1 | NM_021145 | 80.20 |
| hsa-miR-200a-5p | MYBL1 | NM_001080416 | 80.45 | hsa-miR-4279 | IRF2 | NM_002199 | 80.17 |
| hsa-miR-200b-5p | MYBL1 | NM_001080416 | 80.45 | hsa-miR-124-5p | TMEM56 | NM_152487 | 80.17 |
| hsa-miR-4279 | HIPK1 | NM_198269 | 80.42 | hsa-miR-4279 | CACNG8 | NM_031895 | 80.16 |
| hsa-miR-4279 | TCEA1 | NM_201437 | 80.41 | hsa-miR-124-5p | APLN | NM_017413 | 80.09 |
| hsa-miR-8064 | ZBTB20 | NM_001164342 | 80.40 | hsa-miR-4279 | MYO5A | NM_000259 | 80.06 |
| hsa-miR-200a-5p | ZNF770 | NM_014106 | 80.36 | hsa-miR-4279 | PRKAG2 | NM_024429 | 80.06 |
| hsa-miR-200b-5p | ZNF770 | NM_014106 | 80.36 | hsa-miR-4279 | C5orf63 | NM_001164479 | 80.05 |
| hsa-miR-8064 | TDP2 | NM_016614 | 80.30 | hsa-miR-4279 | GPR62 | NM_080865 | 80.03 |
| hsa-miR-124-5p | USP30 | NM_032663 | 80.25 | hsa-miR-200a-5p | PAPOLG | NM_022894 | 80.01 |
| hsa-miR-642b-5p | ARRDC3 | NM_020801 | 80.25 | hsa-miR-200b-5p | PAPOLG | NM_022894 | 80.01 |
